# Method for Assessing the Influence of Phobic Stimuli in Virtual Simulators

**DOI:** 10.3390/jimaging9100195

**Published:** 2023-09-25

**Authors:** Artem Obukhov, Mikhail Krasnyanskiy, Andrey Volkov, Alexandra Nazarova, Daniil Teselkin, Kirill Patutin, Darya Zajceva

**Affiliations:** The Laboratory of Medical VR Simulator Systems for Training, Diagnostics and Rehabilitation, Tambov State Technical University, Tambov 392000, Russia; mikhail.krasnyanskiy@yandex.ru (M.K.); didim@eclabs.ru (A.V.); nazarova.al.ol@yandex.ru (A.N.); dteselk@mail.ru (D.T.); kirill-patutin@mail.ru (K.P.); darya.zajceva.87@mail.ru (D.Z.)

**Keywords:** virtual reality, phobic effects, professional training, virtual simulators, medical data, electroencephalogram processing

## Abstract

In the organizing of professional training, the assessment of the trainee’s reaction and state in stressful situations is of great importance. Phobic reactions are a specific type of stress reaction that, however, is rarely taken into account when developing virtual simulators, and are a risk factor in the workplace. A method for evaluating the impact of various phobic stimuli on the quality of training is considered, which takes into account the time, accuracy, and speed of performing professional tasks, as well as the characteristics of electroencephalograms (the amplitude, power, coherence, Hurst exponent, and degree of interhemispheric asymmetry). To evaluate the impact of phobias during experimental research, participants in the experimental group performed exercises in different environments: under normal conditions and under the influence of acrophobic and arachnophobic stimuli. The participants were divided into subgroups using clustering algorithms and an expert neurologist. After that, a comparison of the subgroup metrics was carried out. The research conducted makes it possible to partially confirm our hypotheses about the negative impact of phobic effects on some participants in the experimental group. The relationship between the reaction to a phobia and the characteristics of brain activity was revealed, and the characteristics of the electroencephalogram signal were considered as the metrics for detecting a phobic reaction.

## 1. Introduction

The modern professional training of personnel can be organized at different levels depending on the characteristics of the work activity, the complexity of the tasks performed, the requirements for the psychological or physical training of employees, as well as the risk of emergency situations [1]. For many organizations, for high-quality training, the use of lecture material and video courses and the testing of theoretical knowledge are sufficient [2]. On the other hand, in industries where there is a risk to human health or life, the precise execution of complex operations and the careful development of practical skills are required; therefore, it is necessary to increase the amount of practical training provided to the employees, which is often difficult to do without stopping the current production process or creating expensive training centers [3,4].

In such situations, modern digital technologies could be a solution to the identified problem. Creating training courses and scenarios using virtual reality technologies is a more economically effective solution, compared to conducting real exercises during the production process or at specially equipped training sites [5]. Despite a number of existing shortcomings in the field of virtual simulators (the lack of scene objects details, the imperfect ergonomics of user scenarios, and the complexity of organizing tactile interaction with the environment), many studies confirm the increasing effectiveness of conducting training in a virtual environment [6,7].

It should be noted that, in a number of areas and training scenarios (for example, fire-fighting or life-saving training), digital solutions still cannot compete with traditional technology for training in real conditions, due to the lack of personal interaction experience and the presence and realism of sensations [8]. However, the search, improvement, and implementation of the latest personnel training methods using digital (including virtual) technologies are necessary, since traditional professional training has a number of limitations caused by its inability to simulate the most dangerous scenarios, its economic costs, and the constant complication of production processes and equipment, which requires prompt solutions to training problems [9].

Thus, one of the options for solving the problem of the professional training of personnel is the creation of virtual simulators with varying degrees of complexity. Such simulators are currently used to train specialists in many industries, as described in [10]. VR technologies allow developers to model emergency situations such as firefighting, evacuation, first aid, etc. [11,12,13]. However, immersing a person in a virtual environment is not enough. An important component of virtual simulators is the subsequent evaluation of the person’s actions and state, as well as the effectiveness of the decisions made in various training scenarios [14,15,16].

Since stress directly affects the quality of performance in professional tasks, the degree of the influence of a person’s emotional state on training effectiveness is of great interest in the development and operation of virtual simulators. Thus, one of the important goals of modern virtual simulators is to practise standard work scenarios, while also evaluating a person’s state, specifically in stressful situations.

Stressful situations include emergency scenarios (fires, equipment failures, evacuations, collapses, etc.) that can be included in professional training [17,18,19,20,21]. It should be noted that, in most cases, a person tends to maintain their composure and act normally in stressful situations. However, the self-control of employees decreases in cases where they are not familiar with the exact plan of action, are not sufficiently informed about the severity of the situation, and do not have access to tools and emergency exits [22]. On the other hand, individual sources of stress can lead to unpredictable reactions in the workplace. This study considers phobic stimuli such as acrophobia (the fear of heights) and arachnophobia (the fear of spiders).

Therefore, a relevant task is to develop approaches to assessing the impact of phobic influences on a person during training in virtual simulators. This allows for the collection and analysis of data on a person’s state in the normal conditions of performing professional tasks, as well as when exposed to a source of phobias. This study considers the development of theoretical foundations for assessing a person’s state using objective criteria, as well as the development of corresponding software tools based on virtual simulators with the integration of phobic disorder sources and equipment for collecting medical data. An electroencephalogram (EEG) is used as a medical data source. The main purpose of this article is to improve the quality of professional training carried out on virtual simulators by taking into account phobic influences.

The contributions of this paper are:

- The formalization and implementation of a method for assessing the impact of phobic influences on a person during professional training in virtual reality, which takes into account the quantitative metrics of exercise performance and characteristics of EEG signals;
- The development of practical approaches for analyzing and processing user data in virtual simulators, including collecting the quantitative metrics of professional task performance quality and extracting additional information from raw EEG signals, using clustering algorithms to separate users into statistically significant subgroups and identify abnormal subgroups predisposed to phobic influences;
- The formulation and testing of hypotheses about the degree of the influence of phobic stimuli on the effectiveness of performing professional tasks on virtual simulators.

The main limitation of the proposed approach is that this study considers a limited number of interaction scenarios (the first focused on the analysis and accuracy of object placement, the second on the speed of the user’s reaction) of virtual scenes and phobic influences (acrophobia and arachnophobia). In addition, to collect data on the human condition, we only used the electroencephalogram and its characteristics. In the future, it is necessary to expand the number of scenarios, bringing them closer to work activities (for example, firefighting, evacuation, first aid) and sources of stress (additional phobias and irritants), and also to add another medical equipment for measuring the pulse, breathing rate, muscle activity, and other physical parameters of a person.

The article has the following structure: Section 1 discusses virtual simulators, analyzes the research in the field of professional training using virtual reality and medical equipment, and puts forward the main research hypotheses and aim; Section 2 presents a method for assessing the impact of phobic effects on a person in virtual reality, the software and hardware used for data collection, and the research scheme; Section 3 includes the results of the experimental research, and their analysis and generalization; and Section 4 includes a discussion of the obtained results, testing of the hypotheses, and conclusions.

### 1.1. Application of Virtual Reality Technologies and Medical Data Analysis in Professional Training

Among virtual simulators, the subject area of software and hardware systems based on the use of virtual reality devices stands out. These technologies allow for the trainee’s concentration on the process of professional training by eliminating distracting factors and providing the maximum level of realistic interaction with virtual objects. Modern virtual simulators, in addition to high-quality and detailed visualization, provide the necessary functional capabilities for collecting data on the process of learning. Along with commonly accepted metrics (time, accuracy, speed, and number of errors), developers of such systems integrate additional equipment to either enhance the user’s immersion in virtual reality or collect additional information.

Examples of devices that increase the user’s immersion in virtual reality include motion capture suits (for accurate body reconstruction in virtual space [23]), treadmills and tracks (for creating physical loads and more natural movement in virtual reality [24,25,26]), and various simulation equipment (for the simulation of specialized equipment usage, such as fire extinguishers, control panels, levers, self-rescuers, etc. [27,28]).

Integrating data collection equipment into virtual complexes is currently actively being developed because, in addition to the objective metrics of exercise performance quality, the physical and psychological state of the trainee is also of great interest. Collecting such characteristics of a person as the pulse, blood pressure, electromyography (EMG), electrocardiogram (ECG), electroencephalogram (EEG), and others allows for a comprehensive assessment of the state, and identifies non-specific reactions of the trainee to events happening in the virtual scene, which may not be detected by external observation or an analysis of exercise performance quality. On the other hand, such a comprehensive analysis allows for the identification of the causes of low-quality exercise performance, which may be caused by hidden physical or psychological illnesses that can be revealed only in stressful situations. Thus, biological feedback is formed in the virtual simulator, allowing for some assessment or command for the simulator based on the current person’s state. The presence of such feedback increases the adaptability of the simulator to the physical or psychological characteristics of the trainee.

Forming such biological feedback is a non-trivial process for virtual simulator developers. It requires the integration of medical equipment, as well as collecting, processing, and analyzing the incoming information. Among the main sources of medical data that can participate in forming biological feedback, the following equipment can be used:

- Pulse meter: Data from these devices in the form of fitness trackers allows for collecting data on the pulse, number of steps, and calorie expenditure. Devices in this format are unobtrusive for the user and provide continuous monitoring for a sufficient amount of time. On the other hand, such devices have low accuracy, inertia, and sensitivity to skin tone and hair coverage. Additionally, most pulse meters have a private interface, which makes data collection and the subsequent transfer to third-party software difficult [29];
- Electrocardiograph: ECG graphs provide access to more detailed information about QRS complexes, which is a more informative source of data on a patient’s heart rate than the aforementioned pulse meters. However, the use of an electrocardiograph by untrained users is a problem due to the difficulties with setting up channels. An incorrect electrode placement can result in incorrect ECG data, which may affect clinical decisions [30];
- Electromyography (EMG): These devices are presented in the form of a bracelet with sensors that record data on a person’s muscle activity. The obtained data are sources of important information about the person’s condition in many areas, such as musculoskeletal rehabilitation, prosthetics, sports, etc. The main difficulty in their use is in the analysis of EMG data, as they contain a large amount of noise and have low reproducibility [31];
- Electroencephalography (EEG): This refers to devices that allow for data collection on a person’s brain activity in conjunction with VR; this technology allows the tracking of brain processes in various scenarios that can be simulated through visual and auditory stimuli. It should be noted that, when wearing a VR helmet over EEG sensors, signal distortion occurs. A partial solution to this problem is the use of special devices to fix the EEG sensors on the head [32]. However, the most common method of working with such data is pre-processing [33].

Given the specificity of the problem under consideration—studying the influence of stress from phobic stimuli on the quality of professional training—the main reactions are observed in the form of changes in brain activity. Thus, EEG is proposed as the main source of medical data when analyzing a person’s reaction to different stimuli in virtual reality.

### 1.2. Analysis of Medical Data for the Presence of Phobic Influences

As previously noted, in stressful situations, unpredictable phobic effects are of importance since, in most cases, they are not taken into account in the framework of labor activities. The human reaction to such impacts may be sharper than the emergency situations modeled in the training program. Therefore, integrating the most common phobic effects into virtual training simulators can increase the predictability of staff actions by evaluating their response to various stimuli, since the correctness of user actions in a stressful situation is the most important result of training.

The problem of determining a person’s emotional activity through recording human physiological signals is a task that can be solved by many approaches. One such approach is using electrodermal activity (EDA). It combines several indicators: skin potential level, skin potential reaction, spontaneous skin potential reaction, skin resistance level, skin resistance reaction, and spontaneous skin resistance reaction. Since the occurrence of the skin electrical activity is caused mainly by the activity of the sweat glands in human skin, which are under the control of the sympathetic nervous system, a relationship is assumed between the level of human fear and the almost immediate reaction of the skin to it. This was confirmed in studies [34,35]. Assessing the respondent’s stress using respiratory monitoring is a modern method, proven in practice in a study [36], where the consistency of the respiratory reaction occurrence with a person’s fear was checked using the galvanic skin response. The method of tracking blood pressure in patients suffering from anxiety, depression, and panic disorders is also worth noting [37]. In the course of a thorough analysis of existing studies, the authors concluded that there is a relationship between increased blood pressure variability and hypertension in people with psychiatric pathologies, including specific phobias. Research is also being conducted in the field of combining ECG signal analysis with machine-learning methods. Thus, the authors of the article [38] developed a stress detection system based on an ECG recording device and a mobile device for monitoring the psychophysiological state of the user. The use of EEG is also a proven approach for recording and recognizing human signals [39]. When using EEG in conjunction with VR, it is possible to simulate a large number of external influences and track how the impact of these influences is reflected in the human brain.

As previously mentioned, EEG is the most preferred source of data for analyzing a person’s response to a stressful situation. Next, the collection, processing, and analysis of EEG data using virtual simulators are considered.

In this study, the electroencephalogram of the company Neuron-Spectre with 21 electrodes is used, where the F electrode records the activity of the frontal parts of the brain, T temporal, C central, P parietal, and O occipital. The placed electrodes record the right and left hemispheres of the brain. FZ, TZ, CZ, PZ, and OZ electrodes are located along the central line of the human head. For the ease of tracking the reactions of the various lobes, a monopolar montage with a CZ central electrode was chosen.

In the analysis of EEG data, especially relevant are the channels located in the occipital and temporal regions, since in these areas are located the visual and vestibular analyzers responsible for visual perception and the assessment of the body position in space. EEG channels are based on the international system 10–20, which provides a standardized method of electrode placement [40]. From the provided list of channels for analysis, the following are suitable:

O1 and O2: These channels correspond to the primary visual cortex of the brain and are especially important for image processing.

OZ: This channel is located above the middle line of the occipital region, between O1 and O2. It represents the middle line of the occipital cortex and is often included in the analysis of visual processing.

P3, P4, P7, and P8: These channels are located above the parieto-occipital sulcus, adjacent to the occipital area. They can record activity associated with both visual processes and attention processes.

PO7 and PO8: These so-called polar channels are located above the rear of the parieto-occipital sulcus, which is involved in the visual processing of a higher order.

By analyzing EEG signals from the electrodes of these channels, various aspects of visual perception can be explored, such as event-related potentials associated with visual stimuli or gamma fluctuations related to visual processing and object recognition.

However, it is important to note that the specific channels of interest may vary depending on the study aim, the experiment plan, and the specific tested hypotheses. Researchers can include additional channels or change their choice according to their specific requirements.

Many studies show that, in healthy subjects at rest, the average total activity in the alpha range is higher in the right hemisphere than in the left [41,42]. This shows the leading role of the right hemisphere in the formation of the alpha rhythm. Functional disintegration through a decrease in the thalamocortical synchronizing system activity and a violation of the brain asymmetry degree is also detected in a number of mental illnesses [43]. A decrease in the alpha frequency range power is also observed in healthy individuals during mental or cognitive stress. This pattern is also observed during waiting or increased attention and may show high mental activity. It shows that low-amplitude EEGs correlate with increased behavioral activity and increased mental excitability, and, in active individuals, the alpha rhythm is lower than in passive individuals [44]. 

Along with alpha-rhythm depression in patients with phobic disorders, there was an increase in the power of beta activity and in the inter-hemispheric asymmetry for the beta rhythm, with a significant predominance in the frontal, temporal, and occipital regions of the right hemisphere. Such desynchronization of the brain electrical activity was previously detected in patients with panic attacks [45]. Increased beta activity in high emotionality, depression, and anxiety states has also been observed in patients with obsessive-compulsive disorder, as well as in children with attention deficit hyperactivity disorder, which was interpreted as a violation of arousal mechanisms. It should be noted that desynchronization, i.e., the replacement of the dominant alpha rhythm with high-frequency beta activity, is generally characteristic of anxiety disorders [46].

Lateralization of brain function refers to unstable differences in the activity of symmetrical formations of the brain; the dominance and the distribution of functions between the hemispheres are individual. This term describes the functional sensory–motor asymmetry profile, the lateral organization profile of the brain, and the individual functional inter-hemispheric asymmetric profile. It is determined by a set of tests that identify the leading arm, foot, eye, or ear. 

Individual asymmetry should be distinguished, in which there is a probability of the predominance of the right and left hemispheres in each person due to genetics. Hemispheric dominance is a dynamic phenomenon; this means that a temporary shift of predominant activity from one hemisphere to another can occur [47], as well as the smoothing of dominance during rest and sleep. Maximum dominance is expressed when performing complex experimental tasks.

Summarizing the analysis, it is impossible to identify some characteristic of EEG on the basis of which it is possible to construct an analysis of the human state. However, an integrated approach, combining different parameters of the EEG signal, allows for the identification of significant differences between people in the process of exposure to phobias in virtual reality.

### 1.3. Purposes of the Study

The purpose of the study is to improve the quality of professional training by developing and applying methods for assessing the impact of phobic effects on humans and improving the accuracy of the detection of stress responses to these exposures for the subsequent minimization of risks in emergencies.

An analysis of existing studies in the field showed that the achievement of the purpose depends on the confirmation of the following three hypotheses:

**H1:**  *Phobic stimuli affect the quality of professional task performance; it is necessary to choose objective criteria for evaluating the performance of exercises and to analyze and objectively assess the impact of different sources of phobias.*

**H2:**  *Phobic stimuli affect the characteristics of EEG signals of brain activity in users. It is necessary to select and implement methods for extracting characteristics from the EEG and then assess the degree of influence of phobic stimuli on these characteristics.*

**H3:**  *EEG data can be used as an objective assessment to identify abnormalities in a group of people for the early diagnosis of phobias or other stress reactions. An automatic and objective evaluation of the presence or absence of a phobic disorder in a person is an urgent task for diagnosis in employment or occupational health certification.*

Thus, it is necessary to implement a method of assessing the influence of phobic effects on a person, conduct its testing, and assess the extent of the phobia impact on the performance quality of professional tasks and the condition of the person. To do this, it is important to use information about brain activity through an electroencephalogram as an instrument for objective assessment.

## 2. Materials and Methods

To confirm the proposed hypotheses, it is necessary to formalize a method of assessing the influence of phobic effects on a person, which will include a set of objective metrics of the professional task performance quality and characteristics of brain activity. Next, it is necessary to select the hardware and develop software that allow for experimental research and data collection. The methodology is completed by a detailed scheme for conducting experimental studies, starting with the data collection and processing and ending with a comparative analysis and statistical evaluation of the results obtained to confirm hypotheses.

### 2.1. Method of Assessing the Influence of Phobic Stimuli in Virtual Reality

To assess the impact of phobic effects on humans in virtual reality conditions, taking into account the experience of previous studies [48] and existing developments in the field of biological feedback integration into fitness systems and complexes, the following method is formulated:

When assessing the impact of the phobia source on a person, researchers cannot rely on subjective metrics since, after some time after the exposure, the survey of respondents may be incorrect due to a change in their emotional state. As part of the proposed assessment method, only objective metrics are selected and measured directly during the phobic exposure.

In the first phase of the method implementation, quantitative metrics of exercise performance in the virtual training system are introduced.

The exercises’ accuracy based on the objects’ positioning is calculated as follows:

(1) $$A=\frac{\sum_{i=1}^{B}\left|x_{i}-x_{i}^{*}\right|}{N}\cdot 100\%,$$

where B is the number of objects placed in the exercise;

N is the number of actions taken by the user in the exercise;

$x_{i}$ is the position of a virtual object selected by the user;

$x_{i}^{*}$ is the position of the object determined by the exercise.

The exercises’ accuracy based on the speed and precision of the reaction is calculated as follows:

(2) $$A=\frac{K}{N}\cdot 100\%,$$

where K is the number of successful and accurate actions with virtual objects.

The duration of the exercise is as follows:

(3) $$T=T_{f}-T_{0},$$

where:

$T_{f}$ is the end of the exercise;

$T_{0}$ is the start of the exercise.

With time and accuracy, the exercise speed can be calculated. Since the variable N in the Formulae (1) and (2) determines the total number of actions, then the speed calculation in general can be written as follows:

(4) $$S=\frac{N}{T}.$$

Within the framework of this survey, biological feedback is of great importance, and a model of the user’s medical metrics is formed. The main source of data is an electroencephalogram (EEG), which allows researchers to record brain activity during training. We identify this as $X=\{X_{c}\}$ set of EEG channels. Each channel contains sequence of values of brain activity: $X_{c}=\{x_{c,}{}_{i}\}$. Then, the variable CH is the total number of EEG channels.

Due to its large size (at a sampling frequency of 500 Hz, one minute of recording contains 30 thousand values per channel), the EEG data are quite difficult to analyze and compare. Therefore, it is further necessary to work out and analyze the EEG in order to obtain aggregated additional information and identify the signs of the data characterizing the EEG signal.

The easiest way to obtain information about the EEG signal is to calculate its amplitude characteristics [49]. As part of this study, the following parameters of the EEG signal were calculated:

The maximum amplitude for each channel $A_{c}^{\max}$ and the average maximum user amplitude $A^{\max}$ are:

(5) $$A_{c}^{\max}=\max\left(x_{c,}{}_{i}|x_{c,}{}_{i}\in X_{c}\right),$$

(6) $$A^{\max}=\sum_{c=1}^{CH}\frac{A_{c}^{\max}}{CH}.$$

The mean amplitude for each channel $A_{c}^{mean}$ and the mean $A^{mean}$ are:

(7) $$A_{c}^{mean}=\frac{\sum_{x_{c,}{}_{i}\in X_{c}}\left(x_{c,}{}_{i}\right)}{\left|X_{c}\right|},$$

(8) $$A^{mean}=\sum_{c=1}^{CH}\frac{A_{c}^{mean}}{CH}.$$

The standard deviation of the amplitude for each channel $A_{c}^{std}$ and the mean standard deviation of the amplitude $A^{std}$ are:

(9) $$A_{c}^{std}=\sqrt{\frac{\sum_{x_{c,}{}_{i}\in X_{c}}{\left(x_{c,}{}_{i}-A_{c}^{mean}\right)}^{2}}{\left|X_{c}\right|-1}},$$

(10) $$A^{std}=\sum_{c=1}^{CH}\frac{A_{c}^{std}}{CH}.$$

However, the amplitude characteristics of the EEG signal may not accurately show the differences between samples within one person and the group due to the individual circumstances of each person. Therefore, the following additional characteristics are introduced, as described below:

The Hurst exponent is a measure of the long-term memory of time series, meaning long-range dependencies in data that are not the result of cycles. The Hurst exponent estimates the self-similarity of a time series by comparing the oscillating structure of a time series with itself, but in smaller fragments divided into consecutive halves. Hurst values range from 0 to 1, with higher values indicating a smoother trend and less volatility [50,51].

The calculation of the Hurst exponent percentage for each c-channel $H_{c}$ and the average Hurst H is as follows:

(11) $$H_{c}=\frac{\log\left(R/S\right)}{\left|X_{c}\right|},$$

(12) $$H=\sum_{c=1}^{CH}\frac{H_{c}}{CH},$$

where R/S is the amplitude ratio between the highest and lowest event (R) divided by the standard deviation found in the series (S).

When $H_{c}$ is less than 0.5, it is understood that the series in question has a tendency toward stability and is expected to continue to oscillate steadily and unpredictably but around a relatively narrow range of values over time. At $H_{c}$ = 0.5, there is statistical uncertainty and a timeline with oscillations known as Brownian motion. When $H_{c}\to 1$ is observed, it is the maximum order, in which the oscillating structure of a series is very similar to itself in all scales. At $H_{c}\to 0$, the data are in a chaotic state, which is not verified by any of the statistical rules [50].

The next characteristic of the EEG signal is the power spectral density (PSD), which shows how the signal power is distributed by frequencies. According to the studying of approaches to EEG analysis to identify the presence of a phobic reaction, it is necessary to analyze the spectral power of alpha, beta, and theta rhythms. In a study [52], when people go into a state of stress, alpha power decreases and beta power increases.

In this study, the generally accepted Welch’s method is used to calculate PSD for each channel $PSD_{c}$ [53,54] and the average PSD:

(13) $$PSD_{c}(h)=\frac{1}{K}\sum_{i=1}^{K}P\left[i\right],$$

(14) $$PSD(h)=\sum_{c=1}^{CH}\frac{PSD_{c}}{CH},$$

where h represents the EEG rhythms of alpha, beta, or theta;

K is number of the involved window in the PSD calculation;

$P\left[i\right]$ is a periodogram calculated on the basis of the square of the absolute value of the samples of the discrete Fourier transform.

Using calculated values of spectral power, researchers can also determine the degree of interhemispheric asymmetry (IHA) using the formula:

(15) $$IHA(h)=\frac{PSD_{r}(h)–\;PSD_{l}(h)}{PSD_{r}(h)+PSD_{l}(h)}\cdot 100\;\%\;,$$

where $PSD_{r}(h)$ is the value of the spectral power of a frequency component of EEG in the right hemisphere $PSD_{r}(h)$ and $PSD_{l}(h)$ is in the left.

The following set of characteristics is based on a coherent EEG analysis to assess the spectral composition similarity of the two derivations. Coherence is a quantitative metric that shows the association of the brain electrical processes and allows one to estimate the degree of synchronization of the EEG frequency components between different sections of the cerebral cortex [55]. Coherence reflects the degree of comparable EEGs’ similarity in the frequency range (i.e., coherence is a fast Fourier-transform cross-correlation), gives information about the stability of the relationship, evaluates the statistical relationship between the corresponding frequencies of the two processes, and has a high sensitivity [56]. The advantage of coherent EEG analysis is its independence from the amplitude of the signal fluctuations from different parts of the brain. The synchronicity of spectra can be quantified through a non-dimensional parameter of similarity, the coherence coefficient. The coefficient of coherence is calculated as a normalized correlation factor between spectra in selected pairs of derivations. The coherence parameter can vary in the range of 1.0 (spectra are identical) to 0 (spectra are different).

The coherence of $C_{x,y}(f)$ in EEG at frequency f is calculated as follows [57]:

(16) $$\left|C_{x,y}(f)\right|=\frac{\left|P_{x,y}{\left(f\right)}^{2}\right|}{P_{x,x}(f)\cdot P_{y,y}(f)},$$

where $P_{x,y}(f)$ is the cross-spectrum density between x и y channels;

$P_{x,x}(f)$ is the auto-spectral density of x;

$P_{y,y}(f)$ is the auto-spectral density of y.

A coherence matrix MC with the size of CH×CH is constructed, for which the average coherence in the average frequency range is calculated for all combinations of channels. Based on the approach used in [58], the upper triangle matrix is formed with non-zero elements equal to the coherence between channels that are statistically reliable. The validity of the coherence coefficient between pairs of electrodes is addressed by conducting surrogate data analysis [59]. Thus, the coherence matrix $MC_{i}$ for the exercise i takes the form:

(17) $$MC_{i}=\left(\begin{array}{ccccc} NaN & C_{1,2}(f) & ... & C_{1,CH-1}(f) & C_{1,CH}(f)\\ NaN & NaN & C_{2,3}(f) & C_{2,CH-1}(f) & C_{2,CH}(f)\\ NaN & NaN & NaN & C_{3,CH-1}(f) & ...\\ NaN & NaN & NaN & NaN & C_{CH-1,CH}(f)\\ NaN & NaN & NaN & NaN & NaN \end{array}\right),$$

where NaN is an undefined value.

As additional metrics in the study, we use:

- Mean matrix of coherence by group (the arithmetic mean of all matrix entries in the group by size G):
  (18) $$\bar{MC}=\sum_{i=1}^{G}MC_{i}/G,$$
- The deviation of the coherence matrix from the mean matrix:
  (19) $$MC_{i}^{SD}=MC_{i}-\bar{MC}.$$

The predetermined quantitative characteristics and metrics for evaluating EEG signals for each exercise allow for an objective comparison of data from each participant in the experimental group, as well as the identification of statistically significant differences between samples from each type of exercise at different exposures in virtual reality.

### 2.2. Software and Hardware for Conducting Research

**Figure 1:**
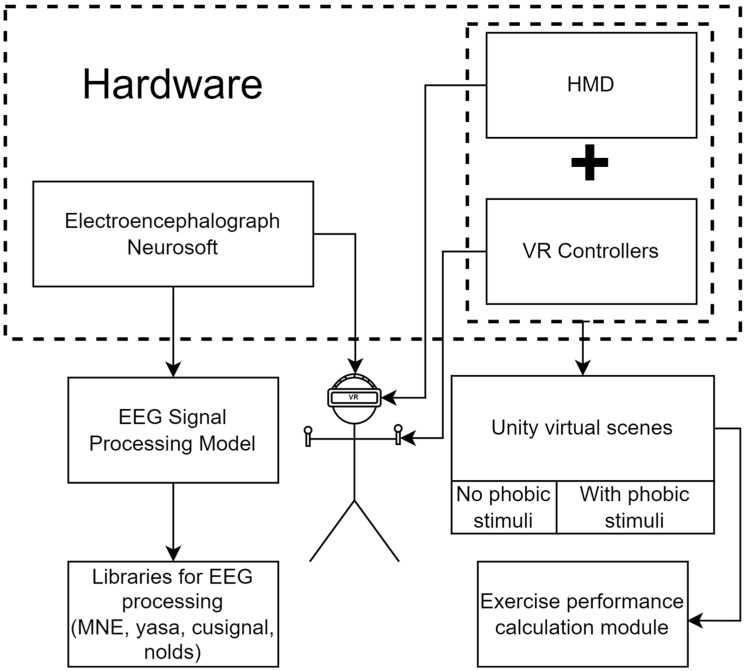
Software and hardware scheme.

For the realization of the above method, it is necessary to provide the software and hardware, the general structure of which is presented in Figure 1.

- The research hardware includes medical equipment for obtaining EEG data and a virtual reality system.
- The electroencephalogram “NEURON-SPECTRUM-4/P” has the following characteristics:
  ◦ 21 EEG channels;
  ◦ Sampling rate: 500 Hz;
  ◦ High-pass filter: 0.5 Hz;
  ◦ Low-pass filter: 70 Hz.

As a virtual reality system, Oculus Quest 2 was used, connected by USB-C interface to a personal computer. The features of the computer include an AMD 16-core CPU, an Nvidia RTX 3060Ti graphics card, 64 GB of RAM, and an SSD drive.

For the successful operation with hardware, it is necessary to develop the appropriate software. This was divided into two groups: virtual scenes for the VR helmet and software modules for data processing.

**Figure 2:**
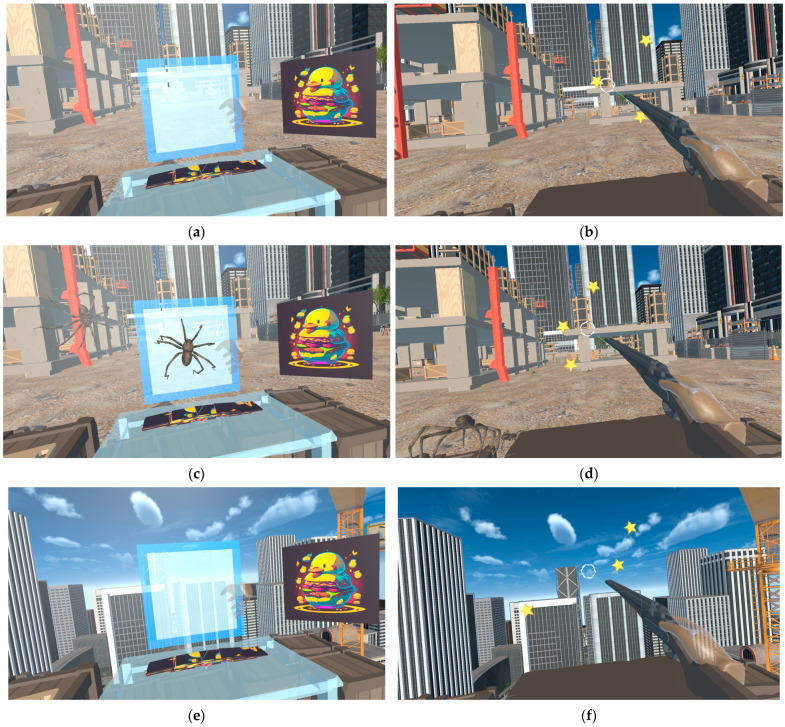
Examples of virtual environments for 6 scenes: (**a**) Puzzle (norm); (**b**) Shooting (norm); (**c**) Puzzle (arachnophobia); (**d**) Shooting (arachnophobia); (**e**) Puzzle (acrophobia); and (**f**) Shooting (acrophobia).

To perform professional tasks, it was necessary to implement basic and additional virtual scenes that include phobic effects (Figure 2). Exercises were the movement of objects (Puzzle) in a given position, which requires attention to the choice of the object being moved and the point of its placement; and shooting at moving objects (Shooting), for example, stars, which allows you to measure the reaction speed and accuracy

At the preliminary stage of research, various exercise options were considered. In the course of analyzing the untrained person’s capabilities in VR and taking into account possible problems with adaptation, control, and complexity of the exercises, it was decided to minimize the exercises’ complexity by focusing on two main areas: an exercise on accuracy and attentiveness (which puzzles satisfy) and an exercise on accuracy and reaction speed (corresponding to shooting). This choice is also due to the fact that many exercises in existing virtual simulators have similar mechanics: human actions when assembling puzzles correspond to exercises for placing objects, interacting with levers, equipment, doors, telephones, etc.; the process of shooting at moving objects is comparable to the actions when using fire extinguishers, selecting objects or buttons using the pointer, and interacting with moving objects [60].

The study examines two common phobias: arachnophobia and acrophobia. The following modifications were made to integrate them into the virtual scene: for arachnophobia, a large number of spiders were placed on the scenes, moving along the surfaces and surrounding the objects and the body of the user; for acrophobia, the activity zone was moved to a significant height above the city.

The reason for choosing arachnophobia was that it is one of the most common animal phobias, along with ophidiophobia [61], and, in addition, during preliminary studies, it created a fairly acute reaction in untrained respondents. Acrophobia was chosen as the most common situational phobia, which is relevant in the framework of our study, since a person is faced with this stressful stimulus in the process of work and everyday activities, and manifestations of symptoms of acrophobia are often present throughout life [62]. The choice of these phobias is also due to the fairly high simplicity of their implementation in virtual space and the absence of the need to create specific conditions. For example, the fear of fire without exposure to warm or hot air cannot be realistically simulated in virtual reality.

These exercise performance metrics and medical data were processed using the following libraries:

- Numpy: For mathematical calculations and matrix processing;
- stats: Provides shapiro method for assessing the normality of data; kruskal for performing the Kruskal–Wallis test; mannwhitneyu for performing the Mann–Whitney U-test;
- nolds: hurst_rs method for calculating the Hurst index
- MNE: Provides classes and methods for the discovery, filtration, and processing of EEG and the calculation of PSD [63];
- Yasa: Method bandpower_from_psd for calculating power by frequency based on PSD;
- cluster: Cluster algorithms KMeans, SpectralClustering, and Birch;
- cusignal: The coherence method for the coherent matrix framework.

### 2.3. Experimental Research Design

Experimental studies were organized according to the following scheme: participants in the experimental group performed the exercises in six virtual scenes in the order indicated in Table 1.

**Figure 3:**
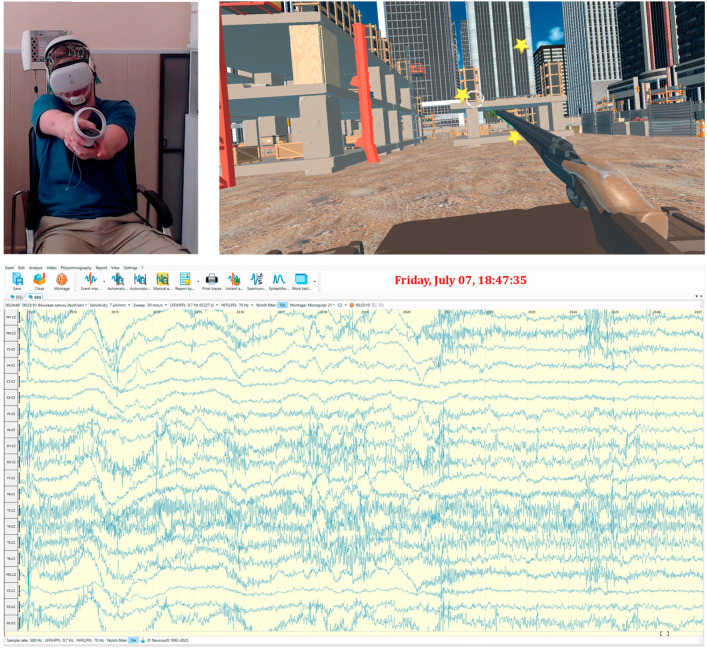
Data collection from virtual reality and medical equipment.

For each of the six scenes, data were collected and processed according to the following algorithm:

1. On the head of each participant were fixed electrodes of the electroencephalograph. Over the electrodes, a virtual reality headset was installed. The general scheme of fixation is presented in Figure 3.
2. Next, software was launched. When analyzing EEG signals, the 18 channels identified earlier were used: FP1, FP2, F3, F4, C3, C4, P3, P4, O1, O2, F7, F8, T3, T4, T5, T6, PZ, and OZ. The exclusion of certain channels is due to the simplification of the response assessment of the right and left hemispheres. If channel CZ is used as the middle electrode, which measures the potential difference of the others, then the potential difference CZ-CZ is equal to zero. The electrodes FPZ and FZ can be neglected, as they were located along the central line of the head, and these channels did not give information from the areas of interest in the study cortex. By analyzing EEG signals from selected channels, it was possible to identify emotions and the processes of a person’s thinking (frontal lobe), reaction to sound stimuli (temporal lobe), visual response and process of recognizing objects (occipital lobe), and processes in the sensory zone of the brain (parietal lobe) [64]. In addition, the use of the listed channels allowed for obtaining a sufficiently complete picture of brain activity to calculate intermembral asymmetry.
3. Human adaptation to virtual reality takes 3–5 min.
4. The respondents performed six exercises with short breaks. The performance of the scenes was recorded with a camera for subsequent verification of human actions. At the same time, video of the virtual scene and a window with the current EEG indications were recorded.
5. Each respondent performed an exercise with the number of actions specified in Table 1 for the subsequent determination of differences in the quantitative metrics of the exercises.
6. The collected data were processed by removing low-quality and noisy areas.
7. Anonymous identifiers were assigned to respondents in the database.
8. Quantitative metrics were calculated by Formulae (1)–(4).
9. The reading of the EEG fragment corresponding to the exercise was carried out using the MNE software library.
10. For each EEG fragment, characteristics were calculated according to the Formulae (5)–(15). The calculation methods and libraries listed in Section 2.2 were used. PSD was calculated separately for alpha, beta, and theta rhythms. The coherence matrices were calculated in accordance with the Expressions (16)–(19).
11. The data were checked for accordance with the normal distribution through the use of the Shapiro–Wilk test, and thermal maps of the variables’ dependence on each other were built, which allowed for finding out the degree of dependency between them.
12. The calculated metrics were combined into comparative tables for each exercise and type of environment, after which the statistical significance of the difference in samples of different scenes was determined by the Kruskal–Wallis method. If such significance was identified, then the statistical difference between individual scenes was further calculated by the Mann–Whitney U-test.
13. Heat maps of the coherence matrix for each user in different scenes were arranged to assess the synchronization of EEG frequency components between different sections of the cerebral cortex in each scene. The average coherence matrices of each scene were compared to each other.
14. To identify differences between users, a vector was formed from the values of time, accuracy, and speed of performance of the exercises and the mean values for the degree of IHA in alpha, beta, and theta rhythms. Vector data were processed by various clustering methods, such as Kmeans, allowing the group members to be distributed into several subgroups.
15. The Kruskal–Wallis test determines the number of clusters (subgroups) for each exercise. A trained cluster algorithm that identified groups with the lowest *p*-value value on quantitative metrics (accuracy, time, and speed of performance of exercises) was preserved.
16. Quantitative metrics and EEG characteristics were analyzed between subgroups for each scene to identify significant differences between them. During the analysis, steps 8–11 were repeated.
17. The degree of IHA for each subgroup was calculated by alpha, beta, and theta rhythms for the corresponding pairs of EEG derivations from the right and left hemispheres: FP2-FP1, F4-F3, C4-C3, P4-P3, O2-O1, F8-F7, T4-T3, and T6-T5.
18. Expert assessment of respondents and their division into normal and abnormal (having a reaction to phobias) subgroups with repetition of steps 8–11. The values of the degree of IHA for the normal and abnormal subgroups were assessed.
19. A summary of the results obtained and an analysis of differences between subgroups were carried out.

Thus, the presented research design allowed researchers to assess the differences between the scenes based on quantitative metrics and the analysis of EEG signals with statistical significance. The tools used allow identification of significant differences between the scenes, the assessment of the presence or absence of phobic influence, and the determination of the most susceptible subgroup among the group of respondents (abnormal).

## 3. Results

In accordance with the experimental design presented above, data were collected from an experimental group of 38 people who were exposed to phobic stimuli. The experimental group has the following characteristics:

- Average age of the group: 20.1 ± 2.1 years;
- The group consists of 32 men and 6 women;
- All respondents do not have diagnosed phobias and have normal or corrected vision;
- The majority (80%) of the group had no experience with interaction with virtual reality.

All participants in the experimental group gave their consent to participate in the research, and for the processing of their personal data.

After a visual analysis of the collected EEG data, due to the large amount of noise, several records were excluded. The data were collected from 28 participants. As a result, 168 records were formed for six scenes, of which 84 relate to the first exercise and 84 to the second. 

### 3.1. Statistical Analysis of Experimental Data

In the first analysis phase, the normality of the distribution of the source data by the main quantitative metrics was assessed. Among the tests for normality, the most common criteria are the normality of Shapiro–Wilk, Kolmogorov, Lilliefors, Anderson–Darling, Kramer–Mises–Smirnov, and others. In this study, the Shapiro–Wilk criteria were chosen for the following reasons: good power characteristics, and high efficiency on small sample sizes (up to 50 elements). The Shapiro–Wilk test was performed using the shapiro function of the SciPy library; an array of metric values for all configurations and attempts was passed to the input of the function. The algorithm of the shapiro function calculates a W statistic that tests whether a random sample comes from (specifically) a normal distribution. Small values of W are evidence of a departure from normality, and percentage points for the W statistic, obtained via Monte Carlo simulation. The Shapiro–Wilk test checks the validity of the null hypothesis: if the null hypothesis is correct, then the data are distributed normally, the alternative hypothesis means that the data do not have a normal distribution [48].

The verification of the data for normality by the Shapiro test showed that the data on the metric of the exercise performance time T are distributed normally with the probability p=0.000, the accuracy of performance A with probability p=0.04, and the speed of performance S with probability p=0.000. Thus, for metrics, the hypothesis of the normal data distribution was not confirmed, so non-parametric tests were used in further analysis.

**Figure 4:**
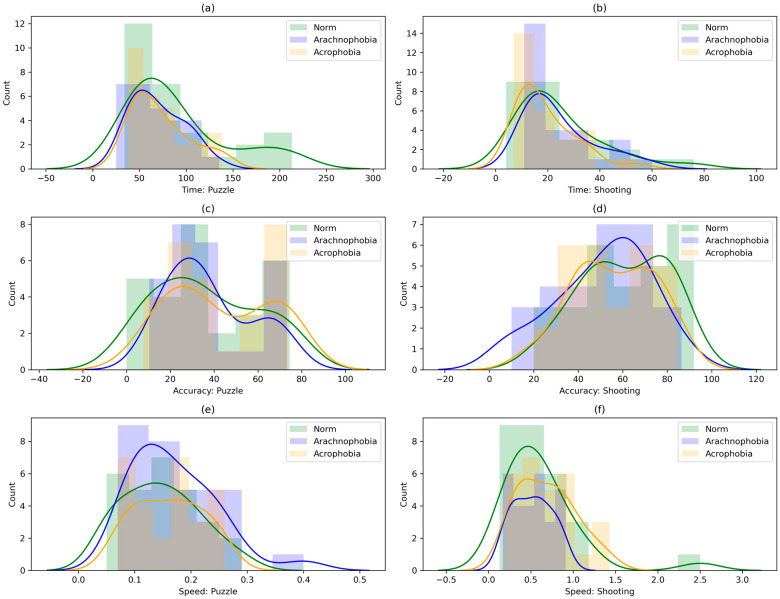
Distribution of data by metrics: (**a**) time for the Puzzle exercises; (**b**) time for the Shooting exercises; (**c**) accuracy for the Puzzle exercises; (**d**) accuracy for the Shooting exercises; (**e**) speed for the Puzzle exercises; and (**f**) speed for the Shooting exercises.

Further, data distribution graphs were built for each metric using the Seaborn library’s function histplot (Figure 4).

**Figure 5:**
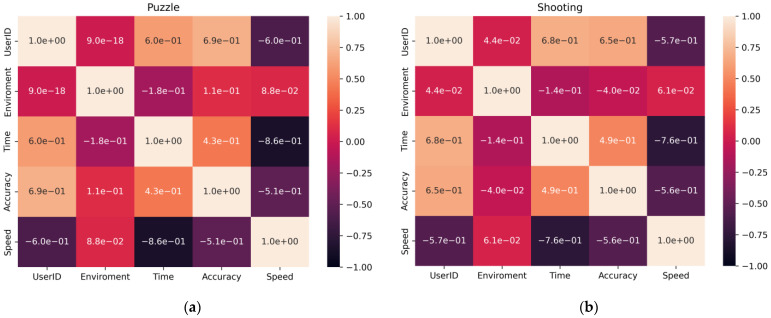
Heatmap of correlation between quantitative metrics of exercises: (**a**) Puzzle; and (**b**) Shooting.

Next, the correlation between the metrics was examined and a heatmap was built for each exercise (Figure 5). 

An analysis of the correlation matrix showed that there were the following dependencies in the data:

Between the environment and precision for the Puzzle exercise: This means that the impact of the phobia affects the accuracy; the environment also influences the time of passage;

Between a specific user and all the quantitative metrics in both exercises, as each user performs the exercise with their own speed, accuracy, and time;

Execution time and accuracy for both exercises, as the speed of the exercise can negatively affect the precision.

Next, the EEG data analysis was conducted. On the evaluation of the initial values of EEG signals, the amplitude characteristics ($A^{\max}$, $A^{mean}$, and $A^{std}$); the PSD value for the alpha-, beta-, and theta rhythms; and the Hurst exponent were obtained. An analysis of the normality data showed that each of the data characteristics on the Shapiro test was p=0.000, which means that the hypothesis of normal data distribution was not confirmed.

**Figure 6:**
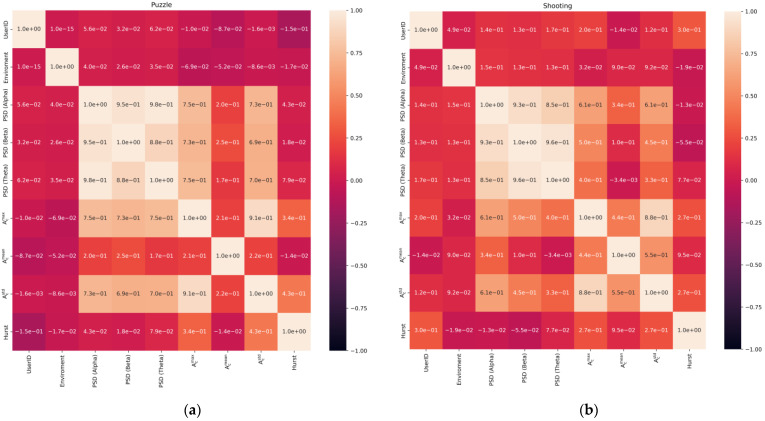
Heatmap of correlation between EEG characteristics: (**a**) Puzzle; and (**b**) Shooting.

The heatmap (Figure 6) is then formed for each exercise. There is some dependence of EEG characteristics on the user ID and the type of environment. There are also logical dependencies between EEG characteristics, so some of them are calculated on the basis of each other.

**Figure 7:**
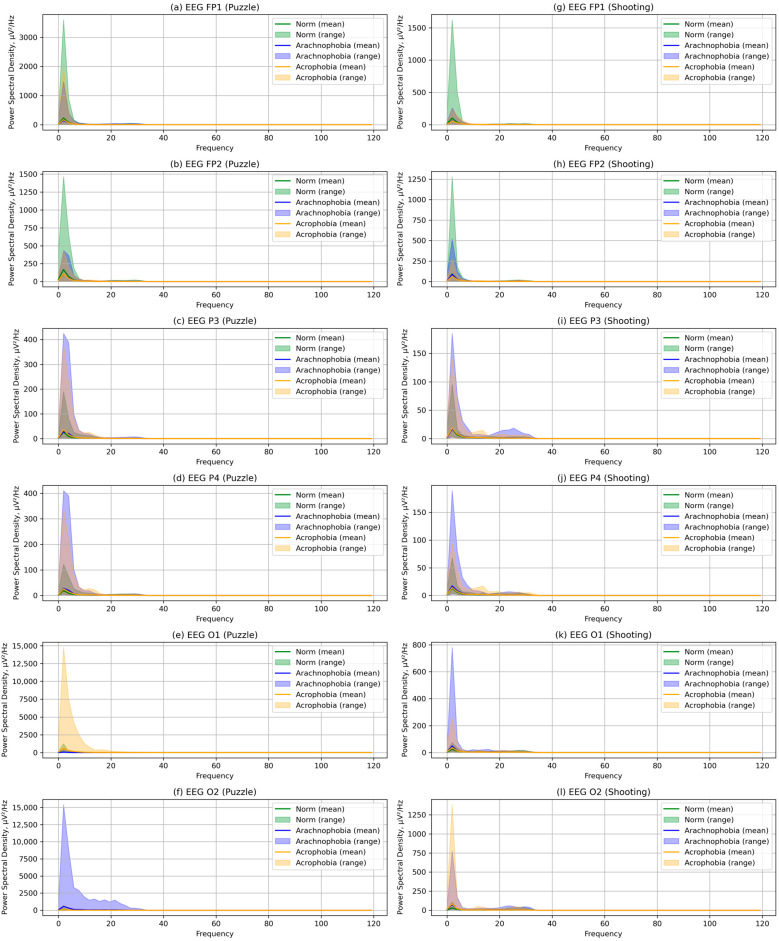
Mean PSD values and the range of their changes by selected channels: (**a**–**f**) Puzzle; and (**g**–**l**) Shooting.

In the course of the collected EEG data analysis, special attention was paid to the EEG channels corresponding to the visual areas of the brain (O1, O2, P3, and P4), as well as the areas responsible for thinking, intellectual activity, fears, and stress (FP1 and FP2). For them, PSD visualization was carried out at frequencies corresponding to the range of 0.4 Hz to 120 Hz, for two exercises and three environments (Figure 7).

The figure shows the mean PSD values, as well as the range of their change from the minimum to the maximum for each frequency. Although the PSD means for the subgroups are similar, the PSD variation ranges vary significantly across some channels. 

Thus, a preliminary statistical analysis of the source data showed that non-parametric tests were necessary in order to compare samples from each scene with each other, and correlation matrices reflect the presence of a certain difference between samples. To determine this difference, a direct comparison of the quantitative metric values and characteristics of EEG was carried out, and the Kruskal–Wallis and Mann–Whitney methods were used to verify the statistical significance of the differences between samples.

### 3.2. Results of Assessment of Phobic Exposure Impact on the Exercises’ Performance

At the next stage of the experimental studies, a comparison of the three environments (norm, arachnophobia, and acrophobia) was carried out in two virtual training exercises. The results of this comparison are presented in Table 2 and Table 3 and contain the mean metric values for each sample with the standard deviation for each scene of the first (Table 2) and second (Table 3) exercises, followed by the probability value p of the Kruskal–Wallis test. If the hypothesis of this test was refuted, then a pair comparison was carried out using the Mann–Whitney U-test. For those pairs of environment types where the value of p is less than 0.05, the corresponding pair of environment identifiers and *p*-value of the Mann–Whitney U-test were indicated.

Generalized values on quantitative metrics and EEG characteristics give ambiguous results. Thus, for performance quality metrics, the difference between samples is not statistically significant (according to the Kruskal–Wallis test). Evaluating the mean metrics values, it can be concluded that the addition of phobic exposure does not have a significant effect on the exercises’ performance time. Moreover, the mean accuracy values show that, in the first exercise (Puzzle), participants took more time to adapt, which led to an increase in the accuracy of the exercises in the second and third scenes even after exposure to the phobia source. Thus, the chosen quantitative metrics do not allow for conclusions about substantial differences between the environments.

On the other hand, metrics related to EEG show significant differences confirmed by the Kruskal–Wallis and Mann–Whitney tests. There is a major measurement of both PSD in the alpha, beta, and theta rhythms, and amplitude characteristics after the addition of phobic effects. The Hurst exponent varies slightly, although statistical tests show a difference between samples. Thus, by changing the characteristics of the EEG between scenes, it is difficult to unambiguously assess the impact of the phobic exposure.

In the comparison of the values obtained, it can be concluded that, in the experimental group, there are some differences between the participants, which, however, cannot be identified when dividing the sample by the types of exercises and environments. Since it is not possible to identify a clear difference between the scenes at this stage, additional analysis of the collected data is necessary. Therefore, the next stage of research is to assess the coherence of the EEG signals. Since the dimensionality of coherence matrices makes their statistical analysis difficult, the studies used an approach based on the visualization of matrices in the form of heatmaps.

**Figure 8:**
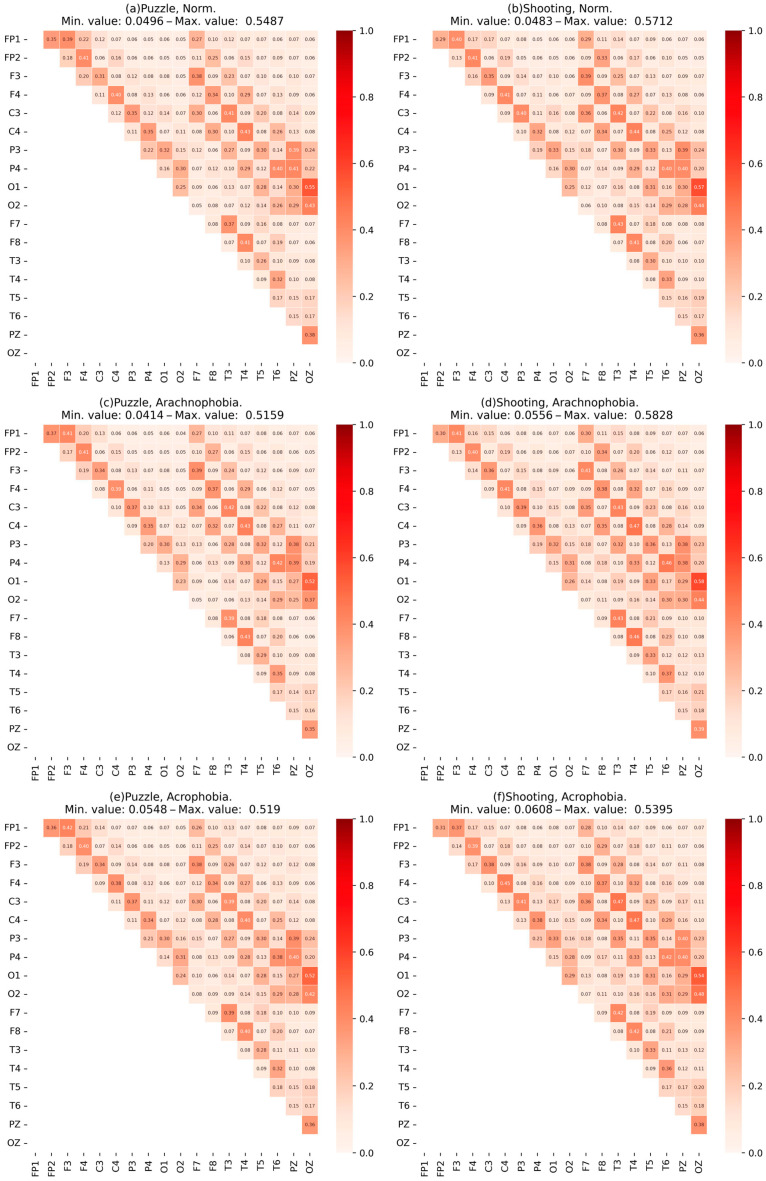
Mean coherence matrices: (**a**) Puzzle, norm; (**b**) Shooting, norm; (**c**) Puzzle, arachnophobia; (**d**) Shooting, arachnophobia; (**e**) Puzzle, acrophobia; and (**f**) Shooting, acrophobia.

Then, for a comparison of the scenes, we calculate and visualize the mean matrix of $\bar{MC}$ coherence for each scene (Figure 8). Each element of the matrix indicates the values of coherence between the respective channels, and the minimum and maximum values are presented in the headline of the matrix among all the channels.

When comparing the mean matrix of coherence values for each scene, significant changes are not observed when adding a phobic effect to the exercise. However, the visual assessment of the coherence matrices of each participant and the calculation of the deviation of these matrices from the mean show significant differences for some subgroups. Thus, it can be concluded that the mean of the scenes does not give the expected result, so it is necessary to further divide the experimental group into subgroups.

### 3.3. Results of Subgroup Analysis Using Cluster Algorithms

To divide the experimental group into subgroups in the absence of the possibility of marking the data, it is necessary to use clustering algorithms. An important benefit is the ability to specify the number of clusters provided by the Kmeans, SpectralClustering, and Birch algorithms of the scikit-learn library.

The starting data used the metrics of accuracy, time, and speed of the exercises, grouped into a vector of nine values (for all three scenes of each exercise). 

To select the optimal algorithm, clustering was carried out by all three methods by varying the number of clusters from two to three, followed by a comparison of how statistically significant, from the point of view of the Kruskal–Wallis test, the difference between these clusters in PSD was. The results of the cluster algorithm comparison are presented in Table 4. Bolded are the results in which the *p*-value of the test between samples is less than 0.05, with an emphasis on selected cluster algorithm variants and optimal cluster sizes.

The testing of algorithms for four, five, or more clusters did not show statistical significance. Additionally, preference was given to the smallest number of clusters when selecting the cluster size and clustering algorithm to increase the number of participants in each subgroup. The average *p*-value for each performance quality metric was also estimated to precisely divide them into subgroups based on exercise effectiveness.

Since almost all variants of divisions of participants in the experimental group were statistically significant in PSD values, the smallest number of clusters was selected. The result is that the optimal number of clusters is equal to two for the first exercise and two for the second.

**Figure 9:**
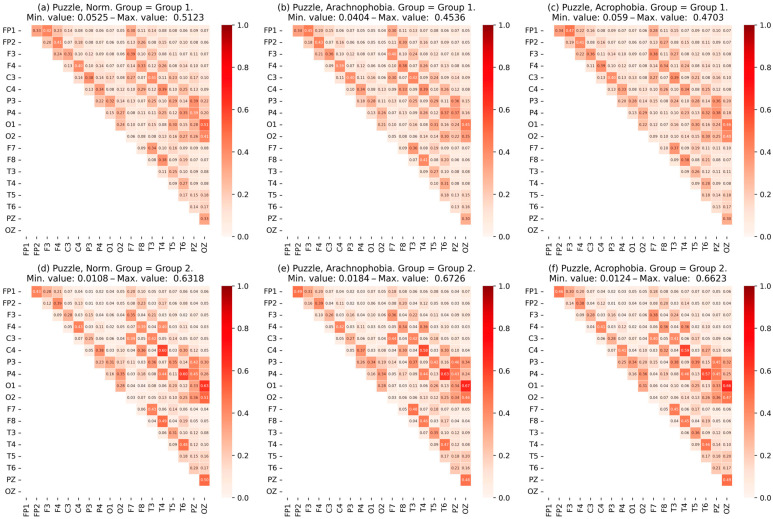
Comparison of the subgroup coherence matrices in each environment of the first exercise: (**a**–**c**) first subgroup; and (**d**–**f**) second subgroup.

**Figure 10:**
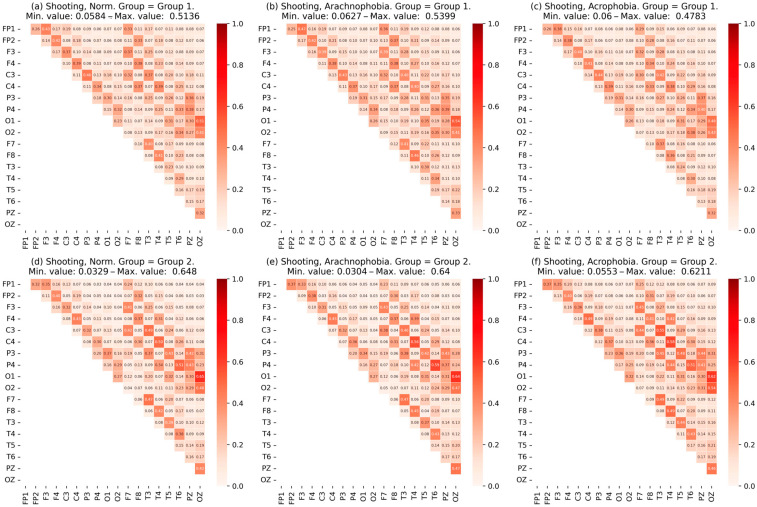
Correlation subgroup coherence matrices in each environment of the second exercise: (**a**–**c**) first subgroup; and (**d**–**f**) second subgroup.

Thus, subgroups were obtained for each exercise. The subgroups were labeled “first” and “second” due to the similarity in group size, which made it difficult to definitively distinguish between normal and abnormal performance. Further, for each subgroup, coherence matrices were built for the first exercise (Figure 9) and for the second exercise (Figure 10).

A visual analysis of the matrix data shows differences in the coherence values between some EEG channels of subgroup participants, as well as in the maximum coherent values. A more detailed analysis of the subgroups’ characteristics is presented further in Section 4.

Due to the presence of clear differences between subgroups in each stage, the next stage of the study requires the calculation of quantitative metrics and EEG characteristics to be repeated, but not between the scenes, as was performed before, but between the subgroups in each scene. The results of this comparison are presented in Table 5 for the first exercise and in Table 6 for the second.

For the verification of the statistical reliability between the metrics of the subgroups using the Kruskal–Wallis test, the *p*-value test is presented in the last column of Table 5 and Table 6. The number of participants for each subgroup is indicated in the last line. Since the data are distributed in different subgroups (the mean values of most characteristics are significantly different), it makes sense to further check the static reliability of the Kruskal–Wallis test between different scenes separately for each subgroup. When indicating the mean metric values for each subgroup, the symbol “*” is additionally assigned if the Kruskal–Wallis test for the subgroup and the metric have a p-value < 0.05. 

At the next stage of the experiment, the degree of IHA for each subgroup was determined. Table 7 shows the IHA values in the first exercise for the normal and abnormal groups; Table 8 shows the values in the second exercise. The asterisk marks the reliability of the differences between the subgroups according to the Mann–Whitney U-test; if the *p*-value is less than 0.05, the symbol “*” is set next to the value. Moreover, for the second group, the corresponding signs are indicated: “↑” if the average IHA values of the other group are greater and “↓” otherwise.

### 3.4. Results of Subgroup Analysis by Expert Assessment Method

During the experimental studies, an expert neurologist was further involved. This specialist visually assessed the reactions of participants in the experimental group, as well as recorded changes in EEG signals. On the basis of his expert assessment, an additional abnormal group consisting of four respondents was identified. Participants in this group showed a reaction to arachnophobia stimuli, which was expressed in tremors in the hands, sharp movements, loud cries, and other non-specific behaviors for the experimental group as a whole. When exposed to acrophobic stimuli, the respondents did not show significant changes in behavior or in EEG data, including the isolated subgroup. However, when comparing coherence matrices for the second exercise, these four respondents were also analyzed as abnormal, although they did not show as many phobic reactions as in the first exercise.

**Figure 11:**
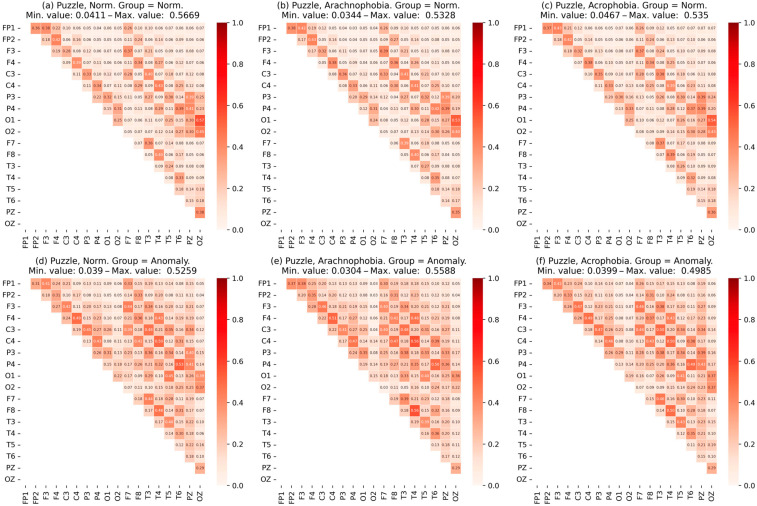
Comparison of the subgroup coherence matrices in each environment of the first exercise: (**a**–**c**) the normal subgroup; and (**d**–**f**) the abnormal subgroup.

**Figure 12:**
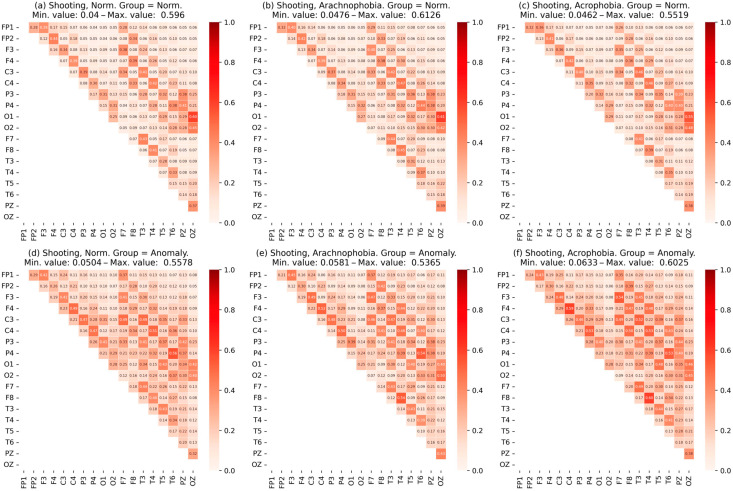
Comparison of the subgroup coherence matrices in each environment of the second exercise: (**a**–**c**) the normal subgroup; and (**d**–**f**) the abnormal subgroup.

Similarly, the normal group and the abnormal group were analyzed. Figure 11 shows the matrix of coherence for subgroup data for the first exercise, Figure 12—for the second exercise.

It can be concluded that the coherence characteristics of an abnormal group have great value. Moreover, it can be noted that this growth is more uniform; i.e., the dependency of the channels in the abnormal group as a whole is higher.

Next, we analyze the performance quality metrics for the two subgroups, as well as the characteristics of the EEG (Table 9).

Finally, the degree of IHA for the normal and abnormal subgroups (Table 10) in two exercises was calculated.

Next, the examination involves analyzing and summarizing the results obtained. An objective assessment of the studies carried out on the basis of the consideration of video materials and the involvement of a doctor for a functional diagnosis will be conducted.

## 4. Discussion

During the comparison of EEG characteristics between scenes, it was found that the addition of phobic stimuli does not have a significant effect on the time, accuracy, or speed of the first exercise (Puzzle). In the second exercise (Shooting), there is a decrease in all metrics under the influence of arachnophobia and their subsequent recovery under the effect of acrophobia. It was also noted that, despite the impact of phobias, the experimental group overall improved performance by adapting to virtual reality. This effect has already been identified in previous studies [48].

A subjective analysis of the experimental group with the involvement of a doctor for a functional diagnosis revealed the following reactions:

- EEG data are very sensitive to movements; in moments of acute human reaction associated with movement, head rotation, shaking, and emotional reflection of what is happening, significant leaps were observed on all EEG channels; similar interference also occurred with sharp movements when performing exercises;
- In the first exercise, many subgroup participants experienced excitement caused by both the non-specific virtual reality environment for them and the need to master ways to interact with it; when spiders appeared, some participants switched to interaction with them, trying to remove them from the working area; some participants were distracted from performing the exercises on the transparent floor with a view from a great height;
- In the second exercise, after adding spiders to the scene, some participants switched their attention to them and began to flinch when interacting with them; at a great height, there was chaotic human interaction with objects and distraction from the surrounding environment.

To identify clearer patterns and differences in the samples, a statistical analysis of EEG characteristics was used, revealing significant differences between the scenes through the Kruskal–Wallis and Mann–Whitney methods (Table 2 and Table 3).

An analysis of the characteristic EEG in the first and second exercises showed that the PSD on the alpha rhythm significantly increases when exposed to arachnophobia and, to a lesser extent, with exposure to acrophobia. The same goes for beta and theta rhythms. The reliability of the changes in amplitude characteristics has been statistically confirmed, but no obvious patterns between exposure to phobias and amplitude values have been identified.

The results of the coherence matrix analysis for all participants in the experimental group, as well as the comparison of the mean coherent matrix for each scene, showed no significant difference. On the other hand, when comparing the matrices of each participant with the mean matrices, certain differences were found: part of the experimental group had significant deviations in coherence values. This allows the researchers to conclude the presence of a certain number of abnormalities—individuals with significant deviations in behavior, exercise metrics, and EEG characteristics relative to the rest of the group. 

Since it was impossible to estimate the size of different abnormal groups in advance, it was proposed that we use clustering algorithms and evaluate the statistical significance of differences between subgroups (clusters) using the Kruskal–Wallis test. The application of the SpectralClustering and KMeans clustering algorithms to data from coherence matrices and IHA degrees allowed the experimental group to be divided into two subgroups within each of the exercises. Group sizes are shown in Table 5 and Table 6. Further analysis of these subgroups revealed the patterns presented below.

### 4.1. Analysis of Automatically Marked Subgroups in the First Exercise

A comparison of the coherence matrices of the subgroups showed certain differences in coherence; for example, in the frontal and temporal lobes, the values in the matrices for the second subgroup were higher. Next, let us perform an analysis of the metrics and characteristics for each of the subgroups.

In the first scene, where the subject did not have phobic effects, the following features were highlighted (Table 5):

- The first subgroup showed less accuracy in the performance of the exercises; such low results may be due to the high speed of action. PSD values for all rhythms were higher than in the second subgroup;
- The second subgroup showed greater accuracy but also a greater performance time. The EEG characteristics of the second group were different: the PSD of all rhythms was lower;
- For the metrics of accuracy, time, speed, and EEG characteristics, a statistically reliable difference was obtained.

In the second scene, a source of arachnophobia was added, which had the following effect on the subgroups:

- The first subgroup slightly reduces accuracy (if the upper limit of values was estimated); PSD values increased significantly at all rhythms, although changes in the amplitude and mean matrix of coherence are not observed;
- The second subgroup improved all metrics: in the PSD, no significant changes were identified, showing the absence of a reaction to arachnophobia; the maximum amplitude of EEG was reduced;
- There was a statistically reliable difference between the performance quality metrics of the exercise, as well as some EEG characteristics.

The third scene had the effect of acrophobia, which also introduced certain changes in the performance of the subgroups:

- The first subgroup showed approximately comparable results with the second scene. The PSD was reduced but remains large enough relative to the first scene;
- The second subgroup further improved the accuracy of execution, but the time increases slightly. Reactions to phobias in the PSD values were not detected;
- Similarly to the second scene, the main metrics were statistically confirmed.

According to the first exercise, the following conclusion can be drawn: Clustering participants with different performance quality, speed, and time, the second group showed clear superiority in the performance of the exercise but without showing any negative reaction to the phobia effects. In contrast, the first group (a sufficiently large number of participants—21) has a reaction to phobic effects, as shown in the PSD values.

Next, the degree of IHA in the first exercise (Table 7) was analyzed. According to an analysis of existing studies, when identifying a phobic reaction in humans, there was a decrease in the activity of the right hemisphere for the frontal (F), occipital (P), and central (C) lobes in the alpha rhythm. In the beta rhythm, there should be a decrease in the activity of the right hemisphere in the temporal (T) and occipital (P) parts, with a possible increase in the frontal, central, and frontal temporal lobes. However, such an effect was not observed in the second group; on the contrary, the results suggested that the first group observed a depression of alpha activity predominantly in the right hemisphere, which led to the functional disintegration of IHA for alpha rhythm, which was stronger in the central occipital lobe. Based on the analysis of existing research in the field of phobia identification and stress, this may indicate a stronger stressful situation for the first group.

### 4.2. Analysis of Automatically Labeled Subgroups in the Second Exercise

The matrix alignment of subgroups in the second exercise shows the difference in the values of coherence across the channels relating to the frontal zone and the occipital zones (Figure 10). The difference between the groups on the matrix of coherence become stronger (the second group had higher values, including the maximum).

It should be noted that more than 50% of the second group was in it again in the second exercise.

The following characteristics of subgroups can be highlighted (Table 6):

- The first group showed less accuracy, but also greater speed, demonstrating a situation similar to the first exercise, as well as higher PSD values;
- The second group again showed greater accuracy through more careful performance of the exercises; PSD values correspond to the previous exercise and were significantly lower;
- The difference between the subgroups in all metrics was statistically reliable.

When added to the second scene of arachnophobic stimuli, the following results were obtained:

- The first subgroup reduced the accuracy and speed of the exercise. PSD values on alpha and theta rhythms increased slightly;
- The second subgroup deteriorated the accuracy metric; PSD increases, significantly stronger than in the first group;
- There was also a statistically reliable difference between the performance quality metrics, as well as some EEG characteristics.

In the third scene, under the influence of acrophobia, the following results were obtained:

- The first subgroup improved relative to the level of the first scene; PSD decreased;
- The second subgroup showed intermediate results, better than the second scene but worse than the first; PSD values remained high;
- The main metrics of the exercise performance were statistically confirmed.

In the second exercise, in both groups, there was a reaction to the phobic effect, leading to a decrease in accuracy, but the amount of the decrease was significantly lower than in the first exercise.

Further analysis of the degree of IHA was conducted in the second exercise (Table 8). The second group observed an increase in the activity of the right hemisphere when exposed to phobias in the frontal (F) and temporal (T) zones. Beta rhythms in the frontal, central, and frontal temporal lobes also increased. Thus, the situation was repeated similarly to the first exercise. This suggests that the second group did not show an aptitude for a phobic reaction. The first group did not have a reaction in the frontal (F), occipital (P), and central (C) areas of the brain in the alpha rhythm. The reason for the lack of a reaction in both groups was the short duration of the exercise and the specifics of the practice itself (part of the participants used a virtual weapon to attack spiders, which allowed them to feel comfortable).

### 4.3. Analysis of Subgroups Labeled by an Expert

Great interest was presented by the comparison of automatically obtained subgroups with manual marking by an expert neurologist. For this purpose, the results presented in Section 3.4 were analyzed.

The coherence matrices (Figure 10) for the abnormal subgroup have a clearly greater mean and a greater correlation between most EEG assignments, despite a slight difference in the maximum values. Moreover, this was observed for all scenes, which indicated the presence of constant stress in respondents even without the influence of phobias.

In the first exercise, increased levels of stress were confirmed by increased PSD values in all scenes, especially the second (when exposed to an individual’s arachnophobic stimuli). A change in all the characteristics of the EEG in a positive direction was observed. Acrophobia did not have such an obvious effect, although there was an increase in PSD.

In the second exercise, the situation repeated itself: the abnormal group had higher PSD values, but, apparently, due to the adaptation to virtual reality and the shorter duration of the exercise, the effect was weaker.

Statistical analysis showed that changes in the PSD and EEG characteristics between scenes for the normal and abnormal groups were statistically reliable.

When analyzing the degree of IHA, the performance of the above conditions for the abnormal subgroup in scenes with arachnophobia was clearly observed: in the alpha-rhythm one, the activity of the right hemisphere decreased, and, in the beta-rhythm one, on the contrary, it increased. This was especially true for the first exercise.

It should be noted that 75% of the abnormalities were automatically grouped into subgroup 1 for the first exercise; for the second exercise, this result is equal to 50%. This shows, on the one hand, the prospect of automatic marking, but, on the other hand, the need to develop more correct clustering algorithms with the capture of more source data and, possibly, their additional processing to improve the accuracy of abnormality recognition. This issue will be the focus of further research.

### 4.4. Argumentation Supporting the Proposed Hypotheses

At the conclusion of the review conducted, the results were analyzed in terms of confirmation or refutation of the proposed hypotheses.

**H1:**  *Phobic stimuli affect the quality of performance of professional tasks.*

The hypothesis was partially confirmed, as, after the automatic division into two subgroups (Section 3.3), within each subgroup, there was a decrease in performance accuracy, especially with arachnophobia. On the other hand, the abnormal group (Section 3.4) having a visual response to arachnophobia did not show any deterioration in the performance of the exercises. However, the source of the phobia acted as a distracting factor, which was noted in the second exercise for many of the study participants.

**H2:**  *Phobic stimuli affect the characteristics of EEG signals and brain activity of users.*

This hypothesis is fully confirmed, as, during the comparison of subgroups in each scene, the average matrix of coherence had significant differences. The automatic and expert division of respondents into subgroups also showed that, for individual subgroups, phobic exposure leads to an increase in values of EEG characteristics.

**H3:**  *EEG data can be used as an objective assessment to identify abnormalities in a group of people for the early diagnosis of phobias or other stress reactions.*

This hypothesis was partially confirmed, as during the processing and analysis of the EEG characteristics given in Table 2 and Table 3, it was not possible to draw a clear conclusion about the user’s reaction. On the other hand, after clustering and dividing the experimental group into subgroups, the change in EEG characteristics became more regular. It has been found that exposure to phobic stimuli leads to an increase in PSD. In the analysis IHA, it was found that respondents prone to phobia showed a decrease in the activity of the right hemisphere in the alpha rhythm and an increase in the beta rhythm.

There was also a change in the mean amplitude of the EEG at the exposure to phobias in the abnormal subgroup. However, the Hurst exponent reflects only the characteristics of the EEG series and does not allow us to draw conclusions about the influence of phobias. The evaluation of the degree of IHA makes it possible to conclude a change in brain activity, but this experiment was only statistically reliable for a limited number of EEG assignments. Thus, among the selected characteristics that are statistically significant and allow us to draw conclusions about the presence of a human reaction to phobic effects are: PSD, coherence matrices, and the degree of IHA.

The division of respondents into two (or more) subgroups using clustering algorithms based on qualitative characteristics and the degree of IHA allowed us to identify certain differences in behavior, after which it is possible to analyze PSD and amplitude characteristics, but, without additional verification (with the involvement of experts), it is impossible to clearly determine whether the chosen subgroup has an increased susceptibility to phobic reaction or stress due to the presence of interference from movement or speech. Thus, EEG should not be the only source of information for the early diagnosis of phobias. This is confirmed by the fact that not all members of an abnormal subgroup fall into one of the subgroups automatically marked by the cluster algorithm.

Research has fully confirmed one of the proposed hypotheses; the remaining two are partially confirmed. The results achieved during the experiments allow us to formulate the following direction for further research: the development of more modern and adaptive virtual training systems with integrated biological feedback. Such software and hardware complexes include not only virtual reality systems to immerse a person in normal and emergency situations, but also feedback modules functioning on the basis of the analysis and processing of medical data (EEG, and, in the future, EMG and ECG). Such integration will allow us to solve, in an automatic mode, the following quite urgent tasks: the assessment of the stress resistance of employees; the flexible regulation of complexity depending on the level of stress or brain activity; the adaptive correction of phobic disorders in virtual reality with the adjustment of the amount of exposure to the source of the phobia.

## 5. Conclusions

The organization of professional training and the assessment of the suitability of employees to perform their work activities is an urgent issue. One of the most important components of this process is to ensure the correct activity of the staff even in stressful conditions, which includes exposure to phobic disorder sources. Even without an obvious predisposition to phobias, an employee under the influence of such a stimulus can make mistakes with a high risk to life or health. It is necessary to assess the risks of this kind of scenario in advance and to identify the impact of the phobic stimuli on the performance quality of professional tasks.

This study presents a method of assessing the influence of phobic effects on a person in virtual reality, aimed at a comprehensive objective assessment of his condition both by quantitative metrics (time, accuracy, and speed of exercises performance) and by assessing the characteristics of his brain activity using EEG data. This approach, in combination with the phobic stimuli in virtual reality, allows for the simulation and evaluation of different scenarios of personnel activity in safe conditions.

In the course of the experimental studies, participants in the experimental group performed exercises both under normal conditions and under exposure to arachnophobia and acrophobia triggers, which changed people’s brain activity. The experiments conducted and analysis of results using the statistical tests Kruskal–Wallis and Mann–Whitney allowed the following conclusion: comparing the metrics of participants in the experimental group in each scene does not allow for objective conclusions about the degree of phobic exposure. The subsequent division of the group into subgroups using clustering algorithms revealed significant differences between the performance quality metrics of professional tasks and the characteristics of the brain activity of subgroup representatives.

An analysis of changes in brain activity when exposed to phobic stimuli led to the following results: The beta rhythm normally characterizes higher nervous activity (cognitive functions and focusing attention) in a normal waking state. When performing or even mentally imagining a movement, the beta rhythm disappears in the zone of the corresponding activity. An increase in the amplitude and index of the beta rhythm is an acute reaction to any stressful influence. The alpha rhythm characterizes a calm, relaxed state of wakefulness, and is best recorded when closing the eyes. Depression occurs when opening his eyes, or when thinking about a task that requires visual representations. A decrease in the amplitude and index of the alpha rhythm, up to a complete disappearance, is typical with an increase in the functional activity of the brain and also for anxiety or fear.

The studies conducted have partially confirmed the hypotheses on the negative impact of phobic effects on some participants in the experimental group, the existence of a relationship between the response to phobias and the characteristics of brain activity, as well as the availability of the prospect of using EEG data as one of the components of a comprehensive assessment for the early diagnosis of phobia disorders.

## Figures and Tables

**Table 1:** Descriptions of virtual scenes.

Scene Number	Exercise Type	Environment Type
1	1. Puzzle (assembling 10 puzzle pieces)	1. Norm
2		2. Arachnophobia
3		3. Acrophobia
4	2. Shooting (10 shots at moving objects)	1. Norm
5		2. Arachnophobia
6		3. Acrophobia

**Table 2:** Comparison of scenes from the first exercise (Puzzle).

Metric	Environment			p-Value (Kruskal–Wallis)	p-Value (Mann–Whitney)
	1. Norm	2. Arachnophobia	3. Acrophobia		
T	**90.321 ± 52.6**	69.679 ± 27.885	72.5 ± 30.291	0.477	-
A	36.54 ± 23.088	37.669 ± 18.741	42.641 ± 22.734	0.593	-
S	0.145 ± 0.067	0.17 ± 0.074	0.16 ± 0.059	0.506	-
PSD (Alpha)	6.678 ± 7.559	22.862 ± 101.149	10.563 ± 28.168	0.681	
PSD (Beta)	20.256 ± 134.652	21.565 ± 171.432	15.368 ± 51.471	0.927	
PSD (Theta)	9.169 ± 11.548	59.8 ± 535.01	15.483 ± 50.036	0.184	
$A^{\max}$	130.619 ± 261.154	113.402 ± 342.393	100.746 ± 95.771	0.0	1–2 (0.0), 1–3 (0.021), 2–3 (0.001)
$A^{mean}$	0.091 ± 0.415	0.157 ± 0.291	0.088 ± 0.323	0.0	1–2 (0.0), 1–3 (0.0), 2–3 (0.0)
$A^{std}$	20.069 ± 18.951	20.869 ± 47.739	18.752 ± 15.571	-	
H	0.873 ± 0.045	0.871 ± 0.043	0.871 ± 0.045	-	

**Table 3:** Comparison of scenes from the second exercise (Shooting).

Metric	Environment			p-Value (Kruskal–Wallis)	p-Value (Mann–Whitney)
	1. Norm	2. Arachnophobia	3. Acrophobia		
T	**24.643 ± 16.395**	24.5 ± 13.276	19.143 ± 11.031	0.175	-
A	60.617 ± 19.131	50.965 ± 20.79	55.814 ± 18.014	0.247	-
S	0.613 ± 0.456	0.518 ± 0.222	0.687 ± 0.33	0.176	-
PSD (Alpha)	4.955 ± 3.679	6.195 ± 8.151	7.688 ± 18.292	0.818	
PSD (Beta)	10.338 ± 49.212	12.02 ± 67.048	10.355 ± 27.168	0.577	
PSD (Theta)	5.847 ± 4.884	9.359 ± 33.705	18.914 ± 99.536	0.0	1–2 (0.0), 1–3 (0.009)
$A^{\max}$	64.476 ± 93.356	74.386 ± 129.27	61.312 ± 47.307	0.001	1–2 (0.0), 2–3 (0.011)
$A^{mean}$	0.047 ± 0.716	0.196 ± 0.942	0.217 ± 1.258	0.0	1–2 (0.0), 1–3 (0.0)
$A^{std}$	14.688 ± 11.75	16.661 ± 18.383	15.52 ± 9.523	-	
H	0.854 ± 0.049	0.855 ± 0.042	0.852 ± 0.05	-	

**Table 4:** Comparison of clustering algorithms.

Exercise	Number of Clusters	Metric	KMeans	Birch	SpectralClustering
Puzzle	2	T	**0.0**	**0.0**	**0.036**
		A	**0.0**	**0.0**	0.848
		S	**0.0**	**0.0**	0.034
	3	T	**0.0**	**0.0**	**0.009**
		A	**0.0**	**0.0**	**0.012**
		S	**0.0**	**0.0**	**0.007**
Shooting	2	T	**0.0**	**0.0**	**0.0**
		A	**0.0**	**0.0**	**0.0**
		S	**0.0**	**0.0**	**0.0**
	3	T	**0.0**	**0.0**	0.102
		A	**0.0**	**0.0**	0.69
		S	**0.0**	**0.0**	0.102

**Table 5:** Comparison of subgroups in different environments of the first exercise (Puzzle).

Environment	Metric	Subgroup		* p-Value
		First	Second	
**1. Norm**	T	65.14 ± 23.31	165.86 ± 42.78 *	**0.0**
	A	29.32 ± 21.14	58.19 ± 12.89	**0.005**
	S	0.17 ± 0.06	0.07 ± 0.02 *	**0.0**
	PSD (Alpha)	13.26 ± 9.64	8.38 ± 3.92	**0.0**
	PSD (Beta)	4.64 ± 3.24	2.78 ± 1.44	**0.0**
	PSD (Theta)	14.05 ± 13.39 *	7.77 ± 3.07 *	**0.0**
	$A^{\max}$	138.42 ± 96.31 *	108.37 ± 70.9 *	**0.0**
	$A^{mean}$	0.02 ± 0.17 *	−0.02 ± 0.05 *	**0.0**
	$A^{std}$	20.96 ± 10.67 *	15.94 ± 6.36 *	**0.0**
	H	0.87 ± 0.04 *	0.86 ± 0.04 *	**0.0**
2. Arachnophobia	T	59.67 ± 22.77	99.71 ± 18.74 *	**0.003**
	A	30.12 ± 13.9	60.33 ± 11.86	**0.001**
	S	0.19 ± 0.07	0.1 ± 0.02 *	**0.002**
	PSD (Alpha)	63.41 ± 171.31	8.74 ± 2.83	**0.0**
	PSD (Beta)	18.7 ± 45.73	2.75 ± 1.08	**0.0**
	PSD (Theta)	90.28 ± 281.38 *	7.42 ± 1.76 *	0.086
	$A^{\max}$	131.44 ± 144.93 *	88.33 ± 26.28 *	0.895
	$A^{mean}$	0.08 ± 0.12 *	0.06 ± 0.08 *	0.234
	$A^{std}$	21.91 ± 18.19 *	15.24 ± 4.0 *	**0.047**
	H	0.87 ± 0.04 *	0.87 ± 0.02 *	0.895
3. Acrophobia	T	59.95 ± 21.79	110.14 ± 18.87 *	**0.001**
	A	34.07 ± 19.65	68.37 ± 5.17	**0.003**
	S	0.18 ± 0.05	0.09 ± 0.02 *	**0.0**
	PSD (Alpha)	25.22 ± 46.12	8.68 ± 3.7	**0.0**
	PSD (Beta)	6.71 ± 10.37	2.75 ± 1.5	**0.0**
	PSD (Theta)	31.56 ± 83.68 *	8.11 ± 2.87 *	**0.012**
	$A^{\max}$	119.6 ± 77.92 *	97.45 ± 50.69 *	**0.005**
	$A^{mean}$	−0.0 ± 0.16 *	−0.02 ± 0.07 *	0.355
	$A^{std}$	20.52 ± 13.51 *	16.21 ± 5.8 *	**0.047**
	H	0.87 ± 0.04 *	0.87 ± 0.03 *	0.086
Number of participants in the subgroup		21	7	

* p-value of the Kruskal–Wallis test between subgroups.

**Table 6:** Comparison of subgroups in different environments of the second exercise (Shooting).

Environment	Metric	Subgroup		* p-Value
		First	Second	
**1. Norm**	T	18.56 ± 15.95	32.75 ± 13.15	0.002
	A	49.54 ± 14.86	75.39 ± 13.32	0.0
	S	0.8 ± 0.51	0.36 ± 0.15	0.002
	PSD (Alpha)	8.71 ± 3.69	7.11 ± 1.83	0.0
	PSD (Beta)	3.72 ± 2.15 *	2.67 ± 1.05 *	0.0
	PSD (Theta)	8.02 ± 3. 9*	7.45 ± 3.33 *	0.165
	$A^{\max}$	70.48 ± 51.35 *	63.42 ± 14.71 *	0.0
	$A^{mean}$	−0.05 ± 0.68 *	0.05 ± 0.32 *	0.0
	$A^{std}$	14.9 ± 6.66 *	14.63 ± 5.36 *	0.064
	H	0.83 ± 0.05 *	0.86 ± 0.02 *	0.0
2. Arachnophobia	T	16.75 ± 5.12	34.83 ± 13.76	0.001
	A	39.06 ± 16.95	66.84 ± 13.58	0.0
	S	0.65 ± 0.17	0.34 ± 0.15	0.001
	PSD (Alpha)	11.51 ± 10.05	10.24 ± 11.08	0.0
	PSD (Beta)	4.8 ± 3.48 *	3.06 ± 2.46 *	0.0
	PSD (Theta)	11.18 ± 9.98 *	10.88 ± 9.77 *	0.105
	$A^{\max}$	75.72 ± 49.48 *	79.81 ± 46.51 *	0.064
	$A^{mean}$	0.33 ± 1.33 *	0.05 ± 0.26 *	0.064
	$A^{std}$	16.77 ± 10.6 *	16.07 ± 6.76 *	0.817
	H	0.82 ± 0.04 *	0.86 ± 0.02 *	0.0
3. Acrophobia	T	12.75 ± 4.71	27.67 ± 11.28	0.0
	A	46.12 ± 14.22	68.74 ± 13.97	0.001
	S	0.88 ± 0.27	0.43 ± 0.21	0.0
	PSD (Alpha)	12.39 ± 16.53	17.94 ± 35.89	0.0
	PSD (Beta)	4.51 ± 3.46 *	8.5 ± 20.38 *	0.0
	PSD (Theta)	9.76 ± 7.35 *	76.09 ± 226.87 *	0.817
	$A^{\max}$	69.93 ± 41.31 *	75.77 ± 41.46 *	0.0
	$A^{mean}$	0.31 ± 1.3 *	0.05 ± 0.62 *	0.487
	$A^{std}$	16.9 ± 9.41 *	17.15 ± 7.78 *	0.064
	H	0.81 ± 0.05 *	0.87 ± 0.02 *	0.0
Number of participants in the subgroup		16	12	

* p-value of the Kruskal–Wallis test between subgroups.

**Table 7:** The degree of IHA in the first exercise.

Exercise/Environment	EEG Derivations	The First Subgroup (21 Participants)			The Second Subgroup (7 Participants)		
		Alpha	Beta	Theta	Alpha	Beta	Theta
Puzzle/ Norm	FP2-FP1	−1.0 ± 25.3	−1.1 ± 28.7	1.7 ± 31.9	−2.4 ± 4.0 ↓	4.5 ± 18.9 ↑	−8.2 ± 13.1 ↓
	F4-F3	−0.4 ± 26.0	1.5 ± 26.9	−6.1 ± 29.7	6.6 ± 13.3 ↑	9.8 ± 18.0 ↑	−0.6 ± 22.8 ↑
	C4-C3	9.5 ± 25.6	6.0 ± 30.0	5.4 ± 29.9	10.1 ± 20.0 ↑	17.8 ± 15.1 ↑	−5.6 ± 17.7 ↓
	P4-P3	−3.2 ± 17.6	−7.2 ± 20.4	1.3 ± 16.2	−0.4 ± 9.2 ↑	0.3 ± 9.9 ↑	−0.9 ± 10.4 ↓
	O2-O1	−3.3 ± 30.1	−2.9 ± 35.9	−2.7 ± 27.2	3.8 ± 25.8 ↑	−9.4 ± 26.6 ↓	2.5 ± 11.2 ↑
	F8-F7	−3.2 ± 32.9	8.1 ± 35.0	−2.1 ± 30.9	10.9 ± 15.5 ↑	25.4 ± 7.7 ↑	12.4 ± 18.0 ↑
	T4-T3	4.4 ± 19.6	5.8 ± 32.1	4.5 ± 22.6	−17.2 ± 18.6 ↓	−15.7 ± 28.0 ↓	−14.0 ± 22.4 ↓
	T6-T5	−5.2 ± 26.4	−11.6 ± 34.0	−5.6 ± 25.0 *	−17.4 ± 22.0 ↓	−17.8 ± 20.2 ↓	−27.1 ± 24.1 * ↓
Puzzle/ Arachnophobia	FP2-FP1	−0.9 ± 20.4	−1.2 ± 20.7	−3.7 ± 23.6	−1.7 ± 3.5 ↓	8.5 ± 22.1 ↑	−8.9 ± 8.5 ↓
	F4-F3	8.3 ± 16.2	7.7 ± 20.8	4.1 ± 18.8	8.9 ± 15.1 ↑	9.4 ± 19.9 ↑	2.9 ± 20.9 ↓
	C4-C3	16.9 ± 32.3	7.0 ± 35.0	9.4 ± 36.0	17.2 ± 20.0 ↑	15.1 ± 15.7 ↑	7.5 ± 19.1 ↓
	P4-P3	−0.5 ± 14.0	−4.9 ± 20.5	−2.2 ± 17.3	0.7 ± 11.6 ↑	3.8 ± 7.2 ↑	2.6 ± 7.7 ↑
	O2-O1	2.4 ± 37.5	2.9 ± 39.5	2.6 ± 32.7	−2.5 ± 24.1 ↓	−12.9 ± 27.4 ↓	7.2 ± 17.5 ↑
	F8-F7	3.4 ± 25.2	7.9 ± 26.4	0.9 ± 27.4	10.1 ± 14.5 ↑	27.1 ± 12.4 ↑	12.4 ± 18.8 ↑
	T4-T3	0.5 ± 21.0	3.5 ± 31.9	−4.9 ± 24.6	−15.2 ± 20.1 ↓	−16.4 ± 28.4 ↓	−10.2 ± 18.4 ↓
	T6-T5	−8.4 ± 23.9	−10.7 ± 32.8	−12.2 ± 24.0	−11.3 ± 20.0 ↓	−15.2 ± 17.0 ↓	−11.2 ± 11.1 ↑
Puzzle/ Acrophobia	FP2-FP1	0.1 ± 16.7	2.8 ± 21.0	−4.5 ± 27.2	2.2 ± 10.7 ↑	12.7 ± 19.5 ↑	−7.1 ± 18.1 ↓
	F4-F3	3.4 ± 17.6	5.0 ± 20.3	1.2 ± 20.2	9.7 ± 13.8 ↑	12.7 ± 18.8 ↑	−1.7 ± 22.8 ↓
	C4-C3	14.3 ± 29.3	5.7 ± 34.3	3.4 ± 39.2	16.9 ± 19.2 ↑	14.0 ± 12.3 ↑	−1.0 ± 20.2 ↓
	P4-P3	3.5 ± 16.0	−2.1 ± 21.1	−1.7 ± 15.6	0.9 ± 13.2 ↓	1.8 ± 9.0 ↑	1.0 ± 10.7 ↑
	O2-O1	−3.8 ± 39.3	−1.3 ± 41.6	−4.5 ± 32.9	−6.6 ± 25.3 ↓	−14.4 ± 24.9 ↓	2.0 ± 12.7 ↑
	F8-F7	−1.6 ± 23.7	13.9 ± 25.3	−5.2 ± 19.5	7.0 ± 14.2 ↑	21.7 ± 14.9 ↑	2.3 ± 18.3 ↑
	T4-T3	1.9 ± 20.2	3.0 ± 33.7	−3.2 ± 24.8	−16.7 ± 23.6 ↓	−15.9 ± 31.8 ↓	−12.6 ± 19.7 ↓
	T6-T5	−5.2 ± 19.8	−10.6 ± 31.2	−6.5 ± 22.4	−12.5 ± 18.5 ↓	−12.0 ± 14.2 ↓	−13.4 ± 14.0 ↓

**Table 8:** The degree of IHA in the second exercise.

Exercise/Environment	EEG Derivations	The First Subgroup (21 Participants)			The Second Subgroup (7 Participants)		
		Alpha	Beta	Theta	Alpha	Beta	Theta
Shooting/ Norm	FP2-FP1	−1.6 ± 21.0	2.0 ± 27.4	−4.2 ± 29.5	5.0 ± 8.5 ↑	7.6 ± 15.5 ↑	1.6 ± 12.5 ↑
	F4-F3	−2.7 ± 26.4	2.0 ± 27.3	−4.8 ± 29.7	11.6 ± 11.6 ↑	13.6 ± 14.3 ↑	5.3 ± 16.9 ↑
	C4-C3	0.3 ± 24.9	−2.9 ± 30.9	−0.4 ± 24.0	12.1 ± 24.4 ↑	15.3 ± 22.6 ↑	4.1 ± 32.4 ↑
	P4-P3	0.4 ± 16.5	−8.7 ± 24.5	−5.2 ± 11.0	0.5 ± 15.5 ↑	1.1 ± 24.0 ↑	−0.9 ± 20.0 ↑
	O2-O1	4.7 ± 31.3	3.6 ± 35.9	−1.5 ± 16.1	−9.6 ± 34.0 ↓	−13.8 ± 35.9 ↓	−6.8 ± 29.2 ↓
	F8-F7	−5.8 ± 29.3	0.2 ± 23.1 *	−6.2 ± 23.1	11.0 ± 20.7 ↑	23.7 ± 24.4 * ↑	11.4 ± 19.0 ↑
	T4-T3	0.3 ± 27.8	−2.2 ± 38.9	−2.2 ± 22.2	−6.9 ± 15.1 ↓	−1.2 ± 31.2 ↑	−5.6 ± 16.8 ↓
	T6-T5	−9.7 ± 23.2	−18.3 ± 28.0	−12.5 ± 26.2	−16.8 ± 21.6 ↓	−15.2 ± 26.6 ↑	−6.7 ± 22.3 ↑
Shooting/ Arachnophobia	FP2-FP1	0.2 ± 18.7	3.3 ± 31.0	1.1 ± 26.8	7.1 ± 7.2 ↑	10.7 ± 17.1 ↑	4.5 ± 17.0 ↑
	F4-F3	6.6 ± 20.8	2.5 ± 28.5	0.6 ± 21.8	11.2 ± 13.2 ↑	13.3 ± 14.5 ↑	5.2 ± 16.7 ↑
	C4-C3	6.0 ± 26.2	−3.5 ± 25.5	−2.7 ± 22.9	15.0 ± 43.0 ↑	15.1 ± 41.9 ↑	3.5 ± 46.5 ↑
	P4-P3	2.8 ± 20.3	−6.6 ± 24.3	−1.1 ± 13.2	5.2 ± 17.0 ↑	4.7 ± 20.6 ↑	−3.0 ± 17.2 ↓
	O2-O1	4.4 ± 28.0	−1.5 ± 35.4	3.1 ± 19.3	3.1 ± 34.7 ↓	−5.4 ± 34.9 ↓	−0.5 ± 31.0 ↓
	F8-F7	4.4 ± 14.9	7.8 ± 15.0 *	−1.4 ± 17.9 *	15.5 ± 21.1 ↑	27.8 ± 19.9 * ↑	15.6± 23.1 * ↑
	T4-T3	−2.2 ± 27.0	−6.8 ± 39.4	−1.2 ± 24.5	−8.5 ± 24.5 ↓	−5.3 ± 35.6 ↑	−7.7 ± 22.1 ↓
	T6-T5	−8.8 ± 26.2	−11.6± 32.4	−11.7 ± 27.2	−6.8 ± 21.0 ↑	−4.4 ± 20.7 ↑	−8.1 ± 12.4 ↑
Shooting/ Acrophobia	FP2-FP1	3.3 ± 17.8	7.9 ± 23.7	−1.8 ± 24.3	4.8 ± 11.4 ↑	5.2 ± 14.4 ↓	0.2± 16.2 ↑
	F4-F3	1.0 ± 15.6	3.1 ± 22.3	−5.8 ± 16.2	12.8 ± 14.1 ↑	11.1 ± 12.1 ↑	4.4 ± 12.8 ↑
	C4-C3	−0.6 ± 25.8 *	−4.8 ± 31.7*	−4.0 ± 28.5	25.0 ± 26.7 * ↑	22.8 ± 24.6* ↑	6.9 ± 28.6 ↑
	P4-P3	3.8 ± 15.5	0.9 ± 21.2	−0.8 ± 16.4	3.3 ± 16.8 ↓	4.4 ± 24.8 ↑	−3.0 ± 8.8 ↓
	O2-O1	2.1 ± 28.4	5.6 ± 31.4	3.3 ± 18.9	−10.3 ± 31.0 ↓	−11.8 ± 34.8 ↓	−8.2 ± 28.9 ↓
	F8-F7	2.5 ± 19.7 *	7.9 ± 22.9	−8.9 ± 26.4 *	21.1 ± 19.2 * ↑	25.5 ± 25.0 ↑	17.4 ± 18.4 * ↑
	T4-T3	−0.0 ± 26.0	−7.4 ± 38.7	−7.4 ± 29.2	−0.0 ± 17.6 ↑	2.4 ± 28.5 ↑	−5.7 ± 18.6 ↑
	T6-T5	−2.7 ± 26.2	−10.6 ± 40.3	−13.7 ± 21.8	−1.8 ± 36.9 ↑	−2.0 ± 34.2 ↑	2.0 ± 33.5 ↑

**Table 9:** Comparison of normal and abnormal subgroups in different environments.

Exercise/Environment	Metric	Subgroup		* p-Value
		Normal	Abnormal	
Puzzle/ Norm	T	96.42 ± 54.1	53.75 ± 15.77	0.088
	A	39.34 ± 23.57	19.76 ± 8.41	0.115
	S	0.14 ± 0.06	0.2 ± 0.05	0.094
	PSD (Alpha)	10.79 ± 5.92 *	19.55 ± 16.42 *	0.0
	PSD (Beta)	3.79 ± 2.6 *	6.47 ± 4.1 *	0.0
	PSD (Theta)	10.8 ± 10.62 *	22.57 ± 14.62 *	0.0
	$A^{\max}$	130.39 ± 95.04 *	134.01 ± 66.85 *	0.743
	$A^{mean}$	−0.02 ± 0.11 *	0.18 ± 0.2 *	0.0
	$A^{std}$	18.95 ± 10.05 *	24.22 ± 8.45 *	0.0
	H	0.87 ± 0.04	0.89 ± 0.03	0.0
Puzzle/ Arachnophobia	T	72.79 ± 28.67	51.0 ± 10.17	0.168
	A	39.54 ± 19.42	26.42 ± 6.95	0.168
	S	0.16 ± 0.08	0.2 ± 0.05	0.167
	PSD (Alpha)	38.79 ± 143.33 *	115.42 ± 172.38 *	0.0
	PSD (Beta)	9.21 ± 30.18 *	47.77 ± 67.65 *	0.0
	PSD (Theta)	60.19 ± 254.07 *	125.81 ± 183.55 *	0.0
	$A^{\max}$	104.78 ± 120.26 *	215.96 ± 128.65 *	0.0
	$A^{mean}$	0.06 ± 0.1 *	0.17 ± 0.12 *	0.0
	$A^{std}$	17.55 ± 14.88 *	36.4 ± 13.84 *	0.0
	H	0.86 ± 0.03	0.9 ± 0.04	0.0
Puzzle/ Acrophobia	T	77.67 ± 29.71	41.5 ± 2.5	0.005
	A	44.68 ± 23.6	30.38 ± 10.05	0.264
	S	0.15 ± 0.05	0.24 ± 0.01	0.005
	PSD (Alpha)	18.33 ± 38.06 *	37.61 ± 50.4 *	0.05
	PSD (Beta)	5.16 ± 9.08 *	9.12 ± 8.98 *	0.0
	PSD (Theta)	23.86 ± 77.05 *	36.74 ± 41.66 *	0.0
	$A^{\max}$	110.48 ± 70.5 *	135.58 ± 81.53 *	0.05
	$A^{mean}$	0.01 ± 0.14 *	−0.09 ± 0.14 *	0.0
	$A^{std}$	17.92 ± 10.37 *	28.58 ± 17.28 *	0.0
	H	0.86 ± 0.04	0.89 ± 0.03	0.0
Shooting/ Norm	T	26.38 ± 16.91	14.25 ± 6.3	0.211
	A	62.8 ± 18.67	47.5 ± 16.39	0.222
	S	0.54 ± 0.3	1.06 ± 0.83	0.211
	PSD (Alpha)	7.19 ± 2.04 *	13.01 ± 3.86 *	0.0
	PSD (Beta)	2.81 ± 1.39 *	6.03 ± 1.81 *	0.0
	PSD (Theta)	6.78 ± 2.7*	13.75 ± 3.06 *	0.0
	$A^{\max}$	63.39 ± 40.45 *	91.85 ± 27.78 *	0.0
	$A^{mean}$	0.01 ± 0.53 *	−0.16 ± 0.7 *	0.513
	$A^{std}$	13.88 ± 6.1 *	20.19 ± 2.54 *	0.0
	H	0.84 ± 0.04 *	0.84 ± 0.05 *	0.022
Shooting/ Arachnophobia	T	26.17 ± 13.62	14.5 ± 2.06	0.07
	A	51.54 ± 21.96	47.5 ± 10.9	0.489
	S	0.49 ± 0.22	0.7 ± 0.1	0.07
	PSD (Alpha)	9.74 ± 8.6 *	18.31 ± 16.36 *	0.0
	PSD (Beta)	3.67 ± 2.98 *	6.35 ± 3.5 *	0.0
	PSD (Theta)	9.43 ± 7.51 *	20.77 ± 15.37 *	0.0
	$A^{\max}$	72.27 ± 38.21 *	108.7 ± 80.08 *	0.0
	$A^{mean}$	0.01 ± 0.28 *	1.43 ± 2.28 *	0.0
	$A^{std}$	14.96 ± 5.77 *	25.54 ± 17.09 *	0.0
	H	0.84 ± 0.03 *	0.83 ± 0.05*	0.743
Shooting/ Acrophobia	T	19.92 ± 11.52	14.5 ± 5.55	0.411
	A	55.12 ± 19.15	60.0 ± 7.07	0.62
	S	0.67 ± 0.34	0.77 ± 0.21	0.411
	PSD (Alpha)	13.09 ± 25.92 *	24.84 ± 29.34 *	0.0
	PSD (Beta)	6.06 ± 14.67 *	7.16 ± 5.38 *	0.0
	PSD (Theta)	41.64 ± 164.09 *	17.48 ± 10.53 *	0.0
	$A^{\max}$	67.84 ± 35.92 *	100.02 ± 58.45 *	0.0
	$A^{mean}$	0.01 ± 0.58 *	1.35 ± 2.1 *	0.0
	$A^{std}$	15.87 ± 7.01 *	23.86 ± 13.67 *	0.0
	H	0.84 ± 0.05 *	0.84 ± 0.06 *	0.513
Number of participants in the subgroup		24	4	

* p-value of the Kruskal–Wallis test between subgroups.

**Table 10:** Degree of IHA for normal and abnormal subgroups.

Exercise/Environment	EEG Derivation	Normal Subgroup (24 Participants)			Abnormal Subgroup (4 Participants)		
		Alpha	Beta	Theta	Alpha	Beta	Theta
Puzzle/ Norm	FP2-FP1	−0.2 ± 20.4	−0.3 ± 26.2	1.7 ± 28.3	−8.4 ± 28.9 ↓	4.0 ± 29.1 ↑	−15.4 ± 27.0 ↓
	F4-F3	0.5 ± 25.4	2.6 ± 26.3	−6.7 ± 29.7	6.2 ± 1.4 ↑	9.7 ± 15.3 ↑	7.6 ± 10.3 ↑
	C4-C3	12.9 ± 23.7	12.1 ± 26.3	7.4 ± 26.5 *	−9.9 ± 18.0 ↓	−10.4 ± 26.8 ↓	−25.8 ± 15.2 * ↓
	P4-P3	−1.6 ± 16.7	−5.3± 19.4	0.4 ± 16.1	−7.8 ± 8.6 ↓	−5.5 ± 13.1 ↓	2.8 ± 4.7 ↑
	O2-O1	−2.6 ± 30.8	−8.1± 34.2	−2.1 ± 25.1	5.1 ± 14.9 ↑	16.8 ± 22.6 ↑	2.8 ± 18.3 ↑
	F8-F7	0.9 ± 32.3	13.5 ± 33.1	3.4 ± 30.5	−3.2 ± 9.1 ↓	6.4 ± 17.3 ↓	−9.9 ± 11.7 ↓
	T4-T3	−1.9 ± 20.0	1.1 ± 32.2	−2.8 ± 21.3	4.5 ± 28.2 ↑	−3.7 ± 34.2 ↓	16.3 ± 31.3 ↑
	T6-T5	−8.3 ± 24.0	−14.8 ± 27.5	−11.0 ± 27.4	−8.1 ± 35.2 ↑	−2.9 ± 46.4 ↑	−11.0 ± 20.0 ↑
Puzzle/ Arachnophobia	FP2-FP1	−0.2 ± 15.2	2.3 ± 20.2	−3.9 ± 21.3	−6.7 ± 28.2 ↓	−5.3 ± 27.0 ↓	−11.9 ± 17.7 ↓
	F4-F3	9.0 ± 17.0	8.8 ± 19.8	3.3 ± 20.2	5.2 ± 5.8 ↓	4.3 ± 24.4 ↓	6.9 ± 13.3 ↑
	C4-C3	21.7 ± 27.2	12.0 ± 30.6	15.4 ± 29.4 *	−11.4 ± 28.3 ↓	−8.8 ± 31.0 ↓	−29.9 ± 22.0 * ↓
	P4-P3	0.4 ± 14.4	−3.4 ± 19.3	−2.9 ± 16.0	−3.7 ± 2.8 ↓	1.8 ± 11.3 ↑	10.4 ± 5.8 ↑
	O2-O1	−3.8 ± 30.3	−8.7 ± 31.9 *	−0.1 ± 24.2	31.5 ± 43.2 ↑	44.6 ± 36.4 * ↑	26.9 ± 45.1 ↑
	F8-F7	5.9 ± 24.6	14.3 ± 25.9	6.6 ± 26.8	0.1 ± 8.7 ↓	2.9 ± 17.1 ↓	−13.3 ± 9.3 ↓
	T4-T3	−2.4 ± 22.4	−0.4 ± 31.7	−6.3 ± 24.6	−9.6 ± 16.7 ↓	−8.2 ± 34.5 ↓	−6.1 ± 13.5 ↑
	T6-T5	−7.8 ± 20.9	−13.9 ± 26.2	−9.7 ± 18.2	−17.1 ± 31.8 ↓	0.9 ± 43.1 ↑	−25.4 ± 32.3 ↓
Puzzle/ Acrophobia	FP2-FP1	1.5 ± 14.6	5.2 ± 21.6	−3.0 ± 25.0	−5.1 ± 19.0 ↓	6.1 ± 17.7 ↑	−17.7 ± 23.1 ↓
	F4-F3	4.7 ± 17.9	6.6 ± 21.6	−1.2 ± 21.1	6.6 ± 8.6 ↑	8.6 ± 8.7 ↑	10.7 ± 16.6 ↑
	C4-C3	18.5 ± 25.5	9.7 ± 30.5	7.6 ± 35.1 *	−6.3 ± 27.0 ↓	−4.1 ± 28.3 ↓	−29.7 ± 14.8 * ↓
	P4-P3	2.8 ± 16.6	−1.7 ± 20.1	−2.0 ± 15.5	3.0 ± 2.7 ↑	2.7 ± 6.9 ↑	4.9 ± 3.7 ↑
	O2-O1	−6.4 ± 38.2	−9.8 ± 38.8	−3.8 ± 31.0	6.9 ± 18.9 ↑	26.8 ± 15.8 ↑	2.4 ± 15.0 ↑
	F8-F7	0.5 ± 22.7	16.7 ± 23.5	−2.4 ± 20.6	0.7 ± 18.0 ↑	10.7 ± 22.1 ↓	−8.4 ± 8.9 ↓
	T4-T3	−2.5 ± 22.5	0.5 ± 34.4	−8.4 ± 20.7	−3.9 ± 22.8 ↓	−14.7 ± 30.3 ↓	11.5 ± 33.4 ↑
	T6-T5	−6.2 ± 17.2	−11.9 ± 25.7	−6.8 ± 19.0	−12.0 ± 30.2 ↓	−5.3 ± 38.0 ↑	−16.3 ± 28.5 ↓
Shooting/ Norm	FP2-FP1	0.2 ± 17.3	5.3 ± 22.2	0.3 ± 23.6	7.1 ± 14.8 ↑	−0.7 ± 28.0 ↓	−13.9 ± 22.1 ↓
	F4-F3	2.7 ± 23.4	6.6 ± 24.7	−1.9 ± 25.4	8.1 ± 15.2 ↑	8.8 ± 12.7 ↑	8.1 ± 24.9 ↑
	C4-C3	8.9 ± 24.0	9.3 ± 26.9	3.3 ± 29.6	−15.6 ± 23.0 ↓	−21.6 ± 27.4 ↓	−9.1 ± 11.0 ↓
	P4-P3	−0.1 ± 16.7	−4.2 ± 25.6	−2.9 ± 16.5	3.3 ± 10.8 ↑	−6.2 ± 18.8 ↓	−5.9 ± 8.4 ↓
	O2-O1	−4.4 ± 32.5	−8.5 ± 34.9	−3.9 ± 23.9	16.6 ± 32.0 ↑	24.0 ± 36.7 ↑	−2.7 ± 14.7 ↑
	F8-F7	−1.4 ± 26.8	10.6 ± 25.6	0.9 ± 23.2	18.5 ± 23.5 ↑	8.0 ± 30.3 ↓	4.3 ± 22.3 ↑
	T4-T3	−1.7 ± 24.9	−0.4 ± 36.6	−6.7 ± 16.2	−9.5 ± 9.7 ↓	−9.7 ± 28.8 ↓	14.5 ± 29.6 ↑
	T6-T5	−11.0 ± 20.2	−16.2 ± 28.1	−8.0 ± 21.0	−23.5 ± 32.4 ↓	−21.6 ± 23.1 ↓	−22.2 ± 38.4 ↓
Shooting/ Arachnophobia	FP2-FP1	2.5 ± 15.3	7.7 ± 25.3	2.5 ± 23.5	7.2 ± 14.3 ↑	−1.1 ± 29.8 ↓	3.0 ± 20.8 ↑
	F4-F3	8.9 ± 16.6	9.6 ± 22.0	1.2 ± 20.9	6.5 ± 24.9 ↓	−7.9 ± 30.0 ↓	10.9 ± 7.8 ↑
	C4-C3	10.9 ± 36.6	5.9 ± 36.5	2.2 ± 36.5	3.2 ± 18.9 ↓	−3.9 ± 20.1 ↓	−13.4 ± 20.4 ↓
	P4-P3	2.4 ± 19.1	−1.9 ± 24.3	−2.9 ± 15.9	12.4 ± 16.0 ↑	−0.8 ± 17.6 ↑	4.4 ± 6.2 ↑
	O2-O1	1.3 ± 31.9	−8.9 ± 34.1 *	1.2 ± 26.5	19.0 ± 19.5 ↑	31.2 ± 18.0 * ↑	4.0 ± 13.1 ↑
	F8-F7	10.2 ± 18.6	17.8 ± 19.7	9.3 ± 20.9 *	2.9 ± 17.5 ↓	7.8 ± 18.9 ↓	−15.1 ± 16.0 * ↓
	T4-T3	−5.6 ± 27.4	−4.5 ± 39.5	−7.2 ± 19.8	−0.7 ± 16.7 ↑	−15.6 ± 23.5 ↓	15.2 ± 34.0 ↑
	T6-T5	−4.9 ± 18.5	−6.4 ± 27.5	−7.1 ± 16.3	−26.6 ± 40.2 ↓	−21.3 ± 29.2 ↓	−28.6 ± 38.2 ↓
Shooting/ Acrophobia	FP2-FP1	5.2 ± 13.9	7.1 ± 20.4	0.7 ± 20.6	−3.7 ± 21.0 ↓	4.4 ± 19.8 ↓	−11.2 ± 21.8 ↓
	F4-F3	6.1 ± 16.8	7.0 ± 19.9	−2.3 ± 16.3	5.4 ± 10.6 ↓	3.6 ± 12.9 ↓	3.7 ± 9.7 ↑
	C4-C3	14.1 ± 28.6	10.0 ± 32.9	3.7 ± 29.1	−11.9 ± 21.2 ↓	−10.9 ± 16.8 ↓	−17.5 ± 20.6 ↓
	P4-P3	2.3 ± 15.8	1.5 ± 23.9	−2.4 ± 13.8	11.7 ± 15.5 ↑	8.0 ± 14.6 ↑	2.6 ± 12.1 ↑
	O2-O1	−6.4 ± 29.8	−9.3 ± 30.5 *	−2.8 ± 25.9	16.0 ± 25.1 ↑	42.8 ± 13.2* ↑	5.1 ± 9.9 ↑
	F8-F7	10.3 ± 22.4	17.3 ± 24.1	5.2 ± 27.4	11.5 ± 15.7 ↑	4.7 ± 29.6 ↓	−15.0 ± 11.1 ↓
	T4-T3	−1.7 ± 22.5	−3.1 ± 35.8	−9.4 ± 22.6	10.2 ± 21.9 ↑	−4.0 ± 29.6 ↓	9.5 ± 33.0 ↑
	T6-T5	−4.7 ± 30.2	−9.7 ± 35.9	−6.3 ± 29.9	11.9 ± 33.5 ↑	9.9 ± 45.7 ↑	−10.9 ± 17.0 ↓

## Data Availability

Datasets available on request form corresponding author only as the data are sensitive and participants may be potentially identifiable.

## References

[1] Barnová S., Duda M., Matulčíková M., Gabrhelová G., Hrivíková T. (2022). Further professional on-the-job training of employees in the digital era. Int. J. Eng. Pedagog..

[2] Chan D.W.M., Lam E.W.M., Adabre M.A. (2023). Assessing the Effect of Pedagogical Transition on Classroom Design for Tertiary Education: Perspectives of Teachers and Students. Sustainability.

[3] Paszkiewicz A., Salach M., Dymora P., Bolanowski M., Budzik G., Kubiak P. (2021). Methodology of Implementing Virtual Reality in Education for Industry 4.0. Sustainability.

[4] Hakami Z. (2021). Comparison between Virtual and Traditional Learning Methods for Orthodontic Knowledge and Skills in Dental Students: A Quasi-Experimental Study. Healthcare.

[5] Dewan M.H., Godina R., Chowdhury M.R.K., Noor C.W.M., Wan Nik W.M.N., Man M. (2023). Immersive and Non-Immersive Simulators for the Education and Training in Maritime Domain—A Review. J. Mar. Sci. Eng..

[6] Makarova I., Mustafina J., Boyko A., Fatikhova L., Parsin G., Buyvol P., Shepelev V. (2023). A Virtual Reality Lab for Automotive Service Specialists: A Knowledge Transfer System in the Digital Age. Information.

[7] Li X., Yi W., Chi H.L., Wang X., Chan A.P. (2018). A critical review of virtual and augmented reality (VR/AR) applications in construction safety. Autom. Constr..

[8] Kamińska D., Sapiński T., Wiak S., Tikk T., Haamer R.E., Avots E., Helmi A., Ozcinar C., Anbarjafari G. (2019). Virtual Reality and Its Applications in Education: Survey. Information.

[9] Alenezi M. (2023). Digital learning and digital institution in higher education. Educ. Sci..

[10] Renganayagalu S.K., Mallam S.C., Nazir S. (2021). Effectiveness of VR head mounted displays in professional training: A systematic review. Technol. Knowl. Learn..

[11] Krasnyanskiy M., Obukhov A., Dedov D. (2021). Formalization of the Burning Process of Virtual Reality Objects in Adaptive Training Complexes. J. Imaging.

[12] Roldán J.J., Crespo E., Martín-Barrio A., Peña-Tapia E., Barrientos A. (2019). A training system for Industry 4.0 operators in complex assemblies based on virtual reality and process mining. Robot. Comput. Integr. Manuf..

[13] Barkokebas R., Ritter C., Sirbu V., Li X., Al-Hussein M. Application of virtual reality in task training in the construction manufacturing industry. Proceedings of the International Symposium on Automation and Robotics in Construction (ISARC 2019).

[14] Marín-Morales J., Llinares C., Guixeres J., Alcañiz M. (2020). Emotion recognition in immersive virtual reality: From statistics to affective computing. Sensors.

[15] Krasnyansky M., Karpushkin S., Popov A., Obukhov A., Dedov D. (2020). Methodology of forming the readiness of miners for work in extreme situations using a training complex. Int. J. Emerg. Technol. Learn..

[16] Löcken A., Golling C., Riener A. How should automated vehicles interact with pedestrians? A comparative analysis of interaction concepts in virtual reality. Proceedings of the 11th International Conference on Automotive User Interfaces and Interactive Vehicular Applications (AutomotiveUI 2019).

[17] Patle D.S., Manca D., Nazir S., Sharma S. (2019). Operator training simulators in virtual reality environment for process operators: A review. Virtual Real..

[18] Farra S.L., Gneuhs M., Hodgson E., Kawosa B., Miller E.T., Simon A., Timm N., Hausfeld J. (2019). Comparative cost of virtual reality training and live exercises for training hospital workers for evacuation. Comput. Inform. Nurs..

[19] Zhu Y., Li N. (2021). Virtual and augmented reality technologies for emergency management in the built environments: A state-of-the-art review. J. Saf. Sci. Resil..

[20] Feng Z., González V.A., Mutch C., Amor R., Rahouti A., Baghouz A., Cabrera-Guerrero G. (2020). Towards a customizable immersive virtual reality serious game for earthquake emergency training. Adv. Eng. Inform..

[21] Mystakidis S., Besharat J., Papantzikos G., Christopoulos A., Stylios C., Agorgianitis S., Tselentis D. (2022). Design, development, and evaluation of a virtual reality serious game for school fire preparedness training. Educ. Sci..

[22] Gantt P., Gantt R. (2012). Disaster psychology: Dispelling the myths of panic. Prof. Saf..

[23] Obukhov A., Dedov D., Volkov A., Teselkin D. (2023). Modeling of Nonlinear Dynamic Processes of Human Movement in Virtual Reality Based on Digital Shadows. Computation.

[24] Krasnyanskiy M.N., Obukhov A.D., Dedov D.L. (2022). Control System for an Adaptive Running Platform for Moving in Virtual Reality. Autom. Remote Control.

[25] Keung C.C.W., Kim J.I., Ong Q.M. (2021). Developing a BIM-based MUVR treadmill system for architectural design review and collaboration. Appl. Sci..

[26] Hooks K., Ferguson W., Morillo P., Cruz-Neira C. (2020). Evaluating the user experience of omnidirectional VR walking simulators. Entertain. Comput..

[27] Obukhov A., Dedov D., Arkhipov A. (2020). Determination of the design parameters of the simulator breathing apparatus for training complexes. IOP Conference Series: Materials Science and Engineering (IOP Publishing 2020).

[28] Delazio A., Nakagaki K., Klatzky R.L., Hudson S.E., Lehman J.F., Sample A.P. Force jacket: Pneumatically-actuated jacket for embodied haptic experiences. Proceedings of the 2018 CHI Conference on Human Factors in Computing Systems (CHI 2018).

[29] Nelson B.W., Low C.A., Jacobson N., Areán P., Torous J., Allen N.B. (2020). Guidelines for wrist-worn consumer wearable assessment of heart rate in biobehavioral research. NPJ Digit. Med..

[30] Bifulco P., Narducci F., Vertucci R., Ambruosi P., Cesarelli M., Romano M. (2014). Telemedicine supported by Augmented Reality: An interactive guide for untrained people in performing an ECG test. Biomed. Eng. Online.

[31] Chowdhury R.H., Reaz M.B., Ali M.A.B.M., Bakar A.A., Chellappan K., Chang T.G. (2013). Surface electromyography signal processing and classification techniques. Sensors.

[32] Tauscher J.P., Schottky F.W., Grogorick S., Bittner P.M., Mustafa M., Magnor M. Immersive EEG: Evaluating electroencephalography in virtual reality. Proceedings of the 2019 IEEE Conference on Virtual Reality and 3D User Interfaces (VR) (IEEE 2019).

[33] Gajbhiye P., Tripathy R.K., Bhattacharyya A., Pachori R.B. (2019). Novel approaches for the removal of motion artifact from EEG recordings. IEEE Sens. J..

[34] Jang E.H., Park B.J., Kim S.H., Chung M.A., Sohn J.H. Correlation between Psychological and Physiological Responses during Fear. Proceedings of the International Joint Conference on Biomedical Engineering Systems and Technologies-Volume 4 (BIOSTEC 2014).

[35] Lin H.P., Lin H.Y., Lin W.L., Huang A.C.W. (2011). Effects of stress, depression, and their interaction on heart rate, skin conductance, finger temperature, and respiratory rate: Sympathetic-parasympathetic hypothesis of stress and depression. J. Clin. Psychol..

[36] Mevlevioğlu D., Murphy D., Tabirca S. Visual respiratory feedback in virtual reality exposure therapy: A pilot study. Proceedings of the ACM International Conference on Interactive Media Experiences (IMX 2021).

[37] Shahimi N.H., Lim R., Mat S., Goh C.H., Tan M.P., Lim E. (2022). Association between mental illness and blood pressure variability: A systematic review. BioMed. Eng. OnLine.

[38] Stein D.J., Aguilar-Gaxiola S., Alonso J., Bruffaerts R., de Jonge P., Liu Z., Caldas-De-Almeida J.M., O’neill S., Viana M.C., Al-Hamzawi A.O. (2014). Associations between mental disorders and subsequent onset of hypertension. Gen. Hosp. Psychiatry.

[39] Tychkov A.Y., Chernyshov D.S., Bofanova N.S., Alimuradov A.K., Ovchinnikov D.L., Sotnikov A.M. Virtual reality implementation for assessment and treatment of phobic anxiety disorders. Proceedings of the 2021 5th Scientific School Dynamics of Complex Networks and their Applications (DCNA), IEEE.

[40] Klem G., Lüders H., Jasper H., Elger C. (1999). The ten-twenty electrode system of the International Federation. The International Federation of Clinical Neurophysiology. Electroencephalogr. Clin. Neurophysiol..

[41] Trajkovic J., Di Gregorio F., Ferri F., Marzi C., Diciotti S., Romei V. (2021). Resting state alpha oscillatory activity is a valid and reliable marker of schizotypy. Sci. Rep..

[42] Petrantonakis P.C., Hadjileontiadis L.J. (2011). A novel emotion elicitation index using frontal brain asymmetry for enhanced EEG-based emotion recognition. IEEE Trans. Inf. Technol. Biomed..

[43] Winterer G., Egan M.F., Rädler T., Hyde T., Coppola R., Weinberger D.R. (2001). An association between reduced interhemispheric EEG coherence in the temporal lobe and genetic risk for schizophrenia. Schizophr. Res..

[44] Haegens S., Cousijn H., Wallis G., Harrison P.J., Nobre A.C. (2014). Inter-and intra-individual variability in alpha peak frequency. Neuroimage.

[45] Gordeev S.A. (2008). Clinical-psychophysiological studies of patients with panic attacks with and without agoraphobic disorders. Neurosci. Behav. Physiol..

[46] Roohi-Azizi M., Azimi L., Heysieattalab S., Aamidfar M. (2017). Changes of the brain’s bioelectrical activity in cognition, consciousness, and some mental disorders. Med. J. Islam. Repub. Iran.

[47] Coan J.A., Allen J.J.B.L., McKnight P.E. (2006). A capability model of individual differences in frontal EEG asymmetry. Biol. Psychol..

[48] Obukhov A.D., Krasnyanskiy M.N., Dedov D.L., Nazarova A.O. (2022). The study of virtual reality influence on the process of professional training of miners. Virtual Real..

[49] Krysko V.A., Papkova I.V., Saltykova O.A., Yakovleva T.V., Pavlov S.P., Zhigalov M.V., Krysko A.V. (2019). Visualization of amplitude-frequency characteristics of EEG of pathological and cognitive functions of the brain from a position of nonlinear dynamics. Proc. J. Phys. Conf. Ser..

[50] Córdova F. (2022). On the meaning of Hurst entropy applied to EEG data series. Procedia Comput. Sci..

[51] Raimundo M.S., Okamoto J. (2018). Application of Hurst Exponent (H) and the R/S Analysis in the Classification of FOREX Securities. Int. J. Model. Optim..

[52] Jokić-Begićm N., Begić D. (2003). Quantitative electroencephalogram (qEEG) in combat veterans with post-traumatic stress disorder (PTSD). Nord. J. Psychiatry.

[53] Shen Z., Li G., Fang J., Zhong H., Wang J., Sun Y., Shen X. (2022). Aberrated multidimensional EEG characteristics in patients with generalized anxiety disorder: A machine-learning based analysis framework. Sensors.

[54] Same M.H., Gandubert G., Gleeton G., Ivanov P., Landry R. (2020). Simplified welch algorithm for spectrum monitoring. Appl. Sci..

[55] Weiss S., Rappelsberger P. (2000). Long-range EEG synchronization during word encoding correlates with successful memory performance. Cogn. Brain Res..

[56] Gunduz A., Principe J.C. (2009). Correntropy as a novel measure for nonlinearity tests. Signal Process..

[57] Schroeter M.L., Bücheler M.M., Müller K., Uludağ K., Obrig H., Lohmann G., Tittgemeyer M., Villringer A., von Cramon D. (2004). Towards a standard analysis for functional near-infrared imaging. NeuroImage.

[58] Chernykh M., Vodianyk B., Seleznov I., Harmatiuk D., Zyma I., Popov A., Kiyono K. (2022). Detrending Moving Average, Power Spectral Density, and Coherence: Three EEG-Based Methods to Assess Emotion Irradiation during Facial Perception. Appl. Sci..

[59] Faes L., Pinna G.D., Porta A., Maestri R., Nollo G. (2004). Surrogate data analysis for assessing the significance of the coherence function. IEEE Trans. Biomed. Eng..

[60] Krajčovič M., Gabajová G., Matys M., Grznár P., Dulina Ľ., Kohár R. (2021). 3D Interactive Learning Environment as a Tool for Knowledge Transfer and Retention. Sustainability.

[61] Polák J., Rádlová S., Janovcová M., Flegr J., Landová E., Frynta D. (2020). Scary and nasty beasts: Self-reported fear and disgust of common phobic animals. Br. J. Psychol..

[62] Eaton W.W., Bienvenu O.J., Miloyan B. (2018). Specific phobias. Lancet Psychiatry.

[63] Gramfort A., Luessi M., Larson E., Engemann D.A., Strohmeier D., Brodbeck C., Hämäläinen M.S. (2014). MNE software for processing MEG and EEG data. Neuroimage.

[64] Liu H., Zhang Y., Li Y., Kong X. (2021). Review on emotion recognition based on electroencephalography. Front. Comput. Neurosci..

